# Supplementary material for: Orphan GPR116 mediates the insulin sensitizing effects of the hepatokine FNDC4 in adipose tissue
Source: Nat Commun. 2021 May 20;12:2999. doi: 10.1038/s41467-021-22579-1 (PMC8137956; doi:10.1038/s41467-021-22579-1)
Supplement: Supplementary file 1 — Supplementary Information [file 41467_2021_22579_MOESM1_ESM.pdf]

Supplementary Information

Orphan GPR116 mediates the insulin sensitizing effects of the hepatokine FNDC4 in adipose tissue

Georgiadi A. et al

Correspondence to Anastasia Georgiadi (email: anastasia.georgiadi@helmholtz-muenchen.de) or Stephan Herzig (email : stephan.herzig@helmholtz-muenchen.de)

Supplementary Note 1

FNDC4 ELISA - FNDC4 quantification in plasma or serum- ELISA Validation

Quantification of circulating sFNDC4 was performed by a competitive ELISA against FNDC4, which is EK-067-90 from Phoenix Pharmaceuticals, according to the manufacturer´s instructions. To verify the specificity of this ELISA we performed the following tests: a) we quantified secreted sFNDC4 from human hepatocyte cell media (HepG2) with siFNDC4 mediated deletion of endogenous FNDC4. HepG2 cells express high levels of FNDC4 (Ct value ≅ 24). First, we confirmed that HepG2 cells secrete sFNDC4 by transiently overexpressing FNDC4 carrying a FLAG tag at the N-terminal (FLAG NTF) (extracellular). We were able to detect FLAG tag in the media, confirming that sFNDC4 is secreted from HepG2 cells (Supplementary Fig.1a). Consistent with the idea that sFNDC4 is released after proteolytic cleavage of FNDC4, we observed a decrease in FLAG signal in the media of HepG2 FNDC4NTF overexpressing cells (Supplementary Fig.1a). Using siRNA against FNDC4 (siFNDC4) we achieved 85% deletion of FNDC4 in HepG2 cells (Supplementary Fig.1b-left panel) and we also measured a corresponding decreased of 76% on sFNDC4 in non-concentrated media using the FNDC4 ELISA (Supplementary Fig.1b-right panel). Furthermore, as shown in Fig.2 we also measured decreased circulating levels of sFNDC4 in mice with liver deletion of FDNC4 (AAVshFNDC4) compared to AAVshControl treated mice. Therefore, we conclude that this ELISA is specifically detecting sFNDC4.

To exclude cross reactivity with the sequence homologue FNDC5/Irisin, we spiked FcsFNDC4, native sFNDC4, FcIrisin and native Irisin in diluent at a final concentration of 80ng/ml and indeed we were able to measure the expected concentration of spiked protein only for rec. FcsFNDC4, sFNDC4, whereas rec.FcIrisin or native Irisin were not detectable (Supplementary Fig.1c). Thus, we confirmed no cross reactivity of this ELISA with Irisin.

In addition, we tested the impact of sample dilution (Supplementary Fig.1d) and matrix interference (Supplementary Fig.1e) on the accuracy of sFNDC4 measurements. In both tests, when absorbance values were within the linear part of the standard curve (Supplementary Fig.1f) we acquired measurements within the acceptable range for accuracy with regard the sample dilution effect (acceptable range: 70%-130%) and the matrix effects (80%-120%) [1].

Of note we observed substantial differences between the quantified levels of sFNDC4 in plasma derived from mouse trunk and tail blood. sFNDC4 quantified from tail plasma was up to 10-fold lower to the levels from trunk blood derived plasma (Supplementary Fig.1g). Similar phenomenon has been previously observed for several cytokines [2].

Supplementary Note 2

Table of correspondence between nM and ng/ml for the doses of rec. FcsFNDC4 used in the manuscript and previous publications on native sFNDC4 functions.

FcsFNDC4		sFNDC4		Fc	
**nM**	**ng/ml**	**nM**	**ng/ml**	**nM**	**ng/ml**
0,001	0,1	0,006	0,1	0,001	0,05
0,01	1	0,1	1	0,01	0,5
0,019	10	0,6	10	0,019	10
0,25	25	1,6	25	0,25	12,5
0,5	50	3,1	50	0,5	25
1	100	6,3	100	1	50
10	1000	62,5	1000	10	500
19	1900	200,0	1900	10	1900
28	2800	400,0	2800	10	2800
100	10000	625,0	10000	100	5000
200	20000	1250,0	20000	200	10000
400	40000	2500,0	40000	400	20000

FcsFNDC4 MW: 100000Da
Fc MW: 50000Da
sFNDC4 MW: 16000Da

Reference list (Supplementary):

1. Andreasson, U. et al. A Practical Guide to Immunoassay Method Validation. Front. Neurol. 6, (2015)

2. Mella, J. R., Chiswick, E. L., King, E. & Remick, D. G. LOCATION, LOCATION, LOCATION: CYTOKINE CONCENTRATIONS ARE DEPENDENT ON BLOOD SAMPLING SITE. Shock Augusta Ga 42, 337–342 (2014).

3. Zimmermann, L. et al. A Completely Reimplemented MPI Bioinformatics Toolkit with a New HHpred Server at its Core. J. Mol. Biol. 430, 2237–2243 (2018)

FNDC4 ELISA specicifity test

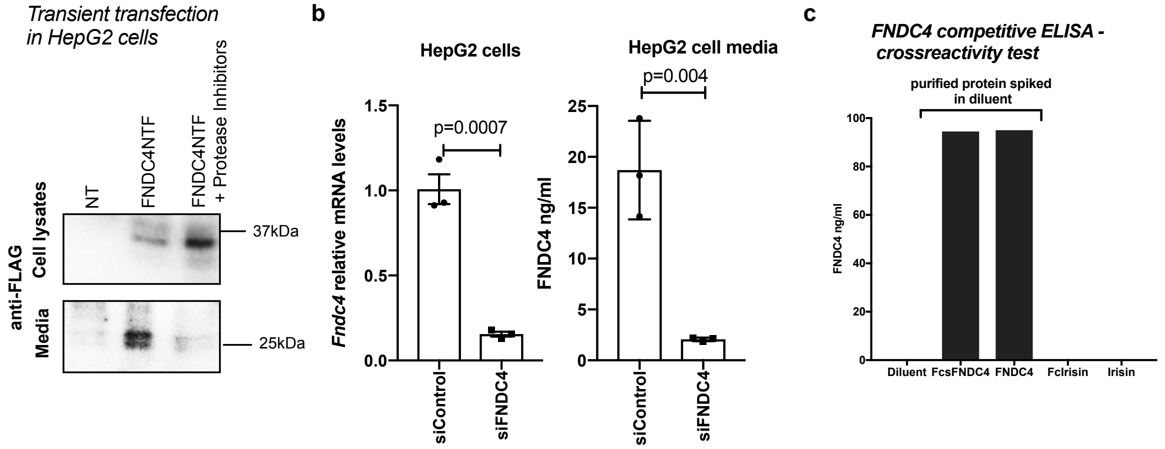

d ***FNDC4 competitive ELISA - Parallelism Assay (effect of dilution on accuracy of measurements)***

mouse plasma	Dilution	OD values	Observed (Extrapolated)-Concentration ng/ml	Adjusted for dilution Concentration ng/ml	Accuracy % (obs/exp)*100
sample 1	1/20	0.8749	6.33 (exp.)	126.63	
	1/80	1.8625	1.34 (obs.)	107.00	84.50
	1/100	2.0086	1.01 (obs.)	101.10	79.84
sample 2	1/20	0.6645	9.39 (exp.)	187.80	
	1/80	1.5061	2.39 (obs.)	190.91	101.66
	1/100	1.7752	1.56 (obs.)	155.68	82.90
sample 3	1/20	1.216	3.69 (exp.)	73.70	
	1/80	2.0786	0.87 (obs.)	69.58	94.41
	1/100	2.0378	0.95 (obs.)	95.10	129.03

e **FNDC4 competitive ELISA - test for matrix effect (spike/recovery test)**

mouse plasma	Dilution	OD vaules	Observed (extrapolated) Concentration ng/ml	Adjusted for dilution Concentration (ng/ml)- Observed Concentratin (ng/ml)	Expected concentated in spiked samped (ng/ml) = concentr. in neat + spiked concentr.	Accuracy % (obs/exp)*100
**neat sample 1**	1/20	0.6645	9.4	**187.80**		
sample 1- spiked 10ng/ml	1/20	0.644	9.8	196.21	197.80	99.197
sample 1- spiked 100ng/ml	1/20	0.462	15.2	304.97	287.80	105.967
**neat sample 2**	1/80	2.079	0.9	**69.58**		
sample 2- spiked 10ng/ml	1/80	1.965	1.1	88.26	79.58	110.910
sample 2- spiked 100ng/ml	1/80	1.740	1.7	132.05	169.58	77.868
**neat sample 3**	1/100	1.775	1.6	**155.68**		
sample 3- spiked 10ng/ml	1/100	1.715	1.7	172.18	165.68	103.925
sample 3- spiked 100ng/ml	1/100	1.618	2.0	201.04	255.68	78.628

f

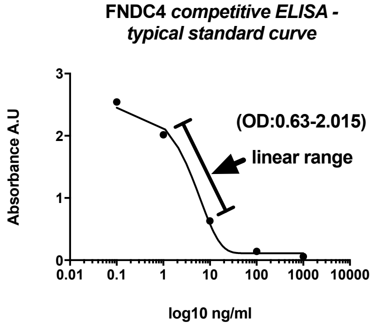

g

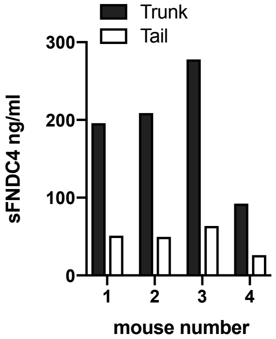

Supplemementary Fig.1 : Specificity and accuracy tests on FNDC4 competitive ELISA. **a** Western blot against the FLAG tag on HepG2 human hepatocytes cell lysates and media (non concentrated). HepG2 cells were tansiently transfected with N-terminal FLAG expressing FNDC4 plasmid (FNDC4 NTF) or lipofectamine control (NT) or with FNDC4 NTF and treated with protease inhibitors to prevent shedding of sFNDC4. This experiment has been performed once on HepG2 cells. **b** RT- quantification of the mRNA of FNDC4 (left panel) and ELISA quantification (right panel) of sFNDC4 in non concentrated media from HepG2 cells transfected with siRNA against FNDC4 (siFNDC4) or siRNA control (siControl). n=3 replicate wells of cells (left panel) and n=3 conditioned media from replicate wells (right panel). Data represent 2^ddCt and *Tbp* is used as a housekeeping gene. **c** ELISA quantification of FNDC4 from left to right: in diluent only, diluent with spiked FcsFNDC4 or FNDC4 (actual spiked amount 80 ng/ml) or FcIrisin or Irisin (actual spiked amount 80 ng/ml). n=1 ELISA well per condition representative of at least 2 experiments . **d** ELISA quantification of FNDC4 in 3 different mouse plasma samples diluted as indicated on the figure. Recovery is calculated based on the 1/20 dilution=expected concentration. **e** ELISA quantification of FNDC4 in 3 different mouse plasma samples, which were spiked with either 10 ng/ml FcFNDC4 or 100 ng/ml FcsFNDC4 and quantified at indicated dilutions. **d,e** this validation analysis was performed once. n=3 representative mice are shown at the table results. **f** Stardard curve of the FNDC4 ELISA used for the calculation of concentrations for (**d**) and (**e**). The linear part of the curve is indicated by an arrow. **g** ELISA quantification of FNDC4 in plasma derived from trunk or tail blood of 4 different mice. Mice were 9-12 weeks old, C57BL6N, males. In **b** data are shown as mean ± SEM, statistics represent unpaired two-tailed t test. Source data are provided as a Source Data file.

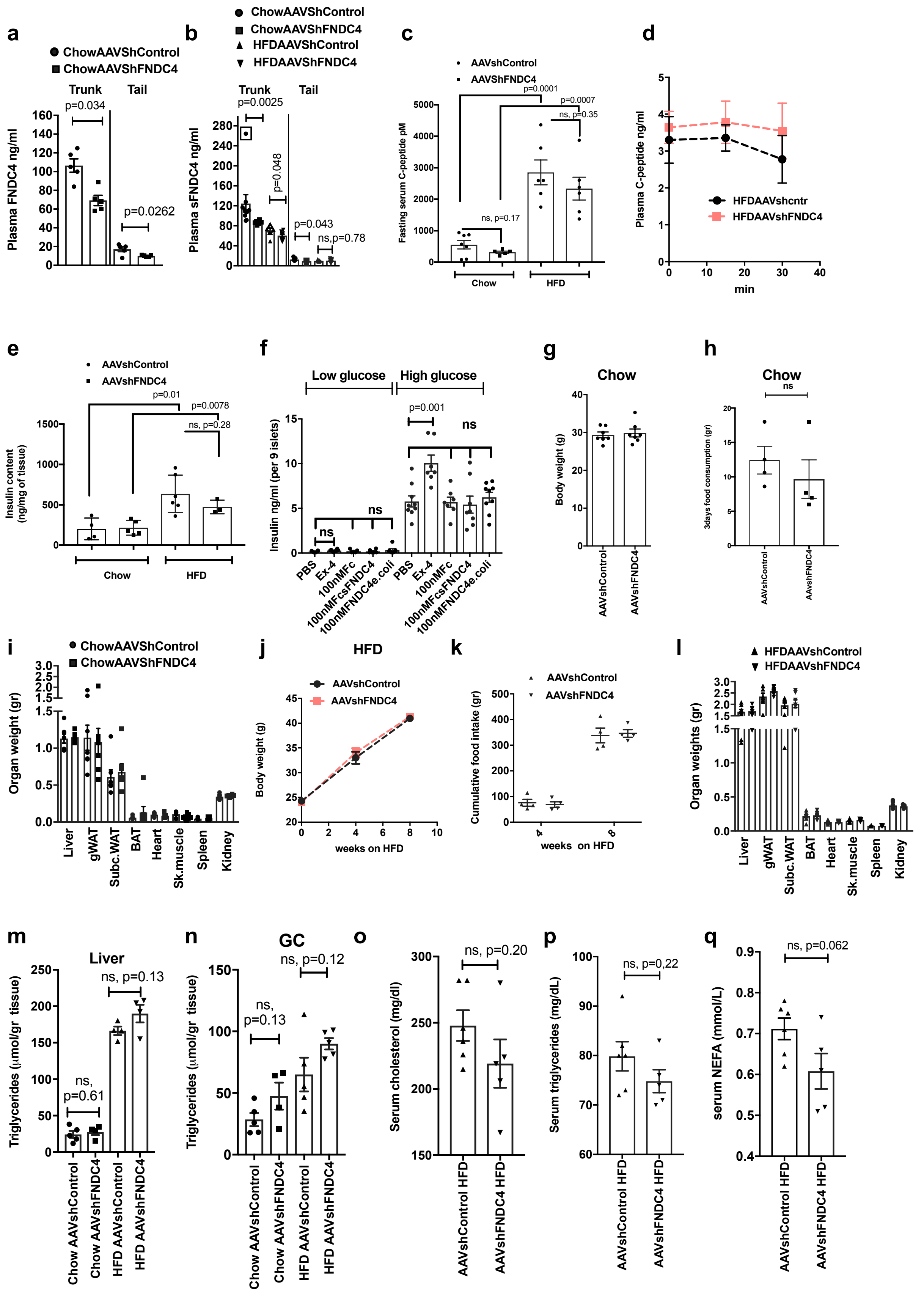

a
ChowAAVShControl
ChowAAVShFNDC4
Trunk
Tail
p=0.034
p=0.0262
Plasma FNDC4 ng/ml
b
ChowAAVShControl
ChowAAVShFNDC4
HFDAAVShControl
HFDAAVShFNDC4
Trunk
Tail
p=0.0025
p=0.048
p=0.043
ns,p=0.78
Plasma sFNDC4 ng/ml
c
AAVshControl
AAVShFNDC4
p=0.0001
p=0.0007
ns, p=0.35
ns, p=0.17
Fasting serum C-peptide pM
Chow
HFD
d
Plasma C-peptide ng/ml
HFDAAVshcntr
HFDAAVshFNDC4
min
e
AAVshControl
AAVshFNDC4
p=0.01
p=0.0078
ns, p=0.28
Insulin content (ng/mg of tissue)
Chow
HFD
f
Low glucose
High glucose
p=0.001
ns
Insulin ng/ml (per 9 islets)
PBS
Ex-4
100nMFc
100nMFcsFNDC4
100nMFNDC4e.coli
g
Chow
Body weight (g)
AAVshControl
AAVshFNDC4
h
Chow
ns
3days food consumption (gr)
AAVshControl
AAVshFNDC4
i
ChowAAVShControl
ChowAAVShFNDC4
Organ weight (gr)
Liver
gWAT
Subc.WAT
BAT
Heart
Sk.muscle
Spleen
Kidney
j
HFD
AAVshControl
AAVshFNDC4
Body weight (g)
weeks on HFD
k
AAVshControl
AAVshFNDC4
Cumulative food intake (gr)
weeks on HFD
l
HFDAAVShControl
HFDAAVshFNDC4
Organ weights (gr)
Liver
gWAT
Subc.WAT
BAT
Heart
Sk.muscle
Spleen
Kidney
m
Liver
ns, p=0.13
ns, p=0.61
Triglycerides (μmol/gr tissue)
Chow AAVshControl
Chow AAVshFNDC4
HFD AAVshControl
HFD AAVshFNDC4
n
GC
ns, p=0.12
ns, p=0.13
Triglycerides (μmol/gr tissue)
Chow AAVshControl
Chow AAVshFNDC4
HFD AAVshControl
HFD AAVshFNDC4
o
ns, p=0.20
Serum cholesterol (mg/dl)
AAVshControl HFD
AAVshFNDC4 HFD
p
ns, p=0,22
Serum triglycerides (mg/dL)
AAVshControl HFD
AAVshFNDC4 HFD
q
ns, p=0.062
serum NEFA (mmol/L)
AAVshControl HFD
AAVshFNDC4 HFD

Supplementary Fig.2 (related to Fig.2): Mice with hepatic deletion of FNDC4 exhibited decreased circulating levels of sFNDC4 and developed a prediabetes phenotype. ELISA quantification of sFNDC4 in plasma from blood collected from trunk or tail of indicated groups at 3 weeks after AAVs injection (**a**) or 11 weeks after AAVs injections (**b**). The ´Trunk´ measurements are also shown in Fig.2, but also included here for comparison purposes. In (**a**) n= 5 mice per group. In (**b**) n=9 mice for TrunkChowAAVshControl, TailChowAAVshControl, Tail HFDAAVshControl and TailHFDAAVshFNDC4, n= 6 mice for TrunkChowAAVshFNDC4, n=8 for TrunkHFDAAVshControl and TrunkHFDAAVshFNDC4, n=7 for Tail ChowAAVshFNDC4. **c** Fasting serum C-peptide (pM) in chow and HFD (8 weeks) fed mice. n=7 mice for ChowAAVshControl, n=5 for ChowAAVshFNDC4 and n= 6 mice for HFDAAVshControl and HFDAAVshFNDC4. **d** Plasma C-peptide (ng/ml) during an IPGTT in mice on HFD for 8 weeks. n=6 mice per group. **e** Insulin content of pancreatic tissue from AAVshFNDC4 and AAVshControl treated mice on chow and HFD for 8 weeks. n=4 mice for ChowAAVshControl, n=5 mice for ChowAAVshFNDC4, n=6 mice for HFDAAVshControl and n=3 mice for HFDAAVshFNDC4. **f** Glucose stimulated insulin secretion from isolated primary islets pre-treated for 24 h with the indicated treatments prior to glucose stimulation. Treatments: Exedin-4 (Ex-4)(a known stimulant of insulin secretion), FcsFNDC4 100 nM (produced in mammalian cells) and FNDC4 e.coli produced (100 nM) and Fc (produced in mammalian cells)100 nM (used as control for the FcsFNDC4 treatment). n=6 replicate wells of 9 mouse islets each, for PBS (low glucose) and 100nMFcsFNDC4 (low glucose), n= 8 replicate wells of 9 mouse islets each, for Ex-4 (low glucose), Ex-4 (high glucose) and 100nMFcsFNDC4(High glucose), n=5 replicate wells of 9 mouse islets each for 100nMFc (low glucose), n=9 replicate wells of 9 mouse islets each, for 100nMFNDC4e.coli (low glucose), PBS(high glucose), 100nMFNDC4e.coli (high glucose), n=7 replicate wells of 9 mouse islets each, for 100nMFc(high glucose). **g** Body weight (n=7 mice per group), **h** food intake, n=4 cages of 2 mice each, **i** organ weight of chow fed control mice (n=7 mice per organ, per group) and **j** body weight, n=8 mice for HFDAAVShControl, n=8 mice for HFDAAVshFNDC4, **k** food intake (n=4 cages of 4 mice each, per group), **l** organ weights for 8 weeks HFD fed mice (n=8 mice per organ, for HFDAAVshControl, n=7 mice per organ, for HFDAAVshFNDC4) . **m** Liver (n=5 mice for ChowAAVshControl, n=4 mice for ChowAAVshFNDC4, HFDAAVshControl, HFDAAVshFNDC4) and **n** skeletal muscle- gastrocnemius (GC) triglyceride content (n=5 mice

for ChowAAVshControl, HFDAAVshControl, HFDAAVshFNDC4 and n=4 mice for ChowAAVshFNDC4), **o** serum cholesterol, **p** serum triglycerides and **q** serum non-esterified fatty acids (NEFA) at indicated groups For **o**,**p**,**q** (n=6 mice for HFDAAVshControl and n=5 mice for HFDAAVshFNDC4). HFD is 45% fat. In all panels bars shown are mean ± SEM. In **a**,**b**,**c**,**e**,**f**,**h**, **m-q** Statistics represent unpaired two-tailed t test. ns; non-significant. Source data are provided as a Source Data file.

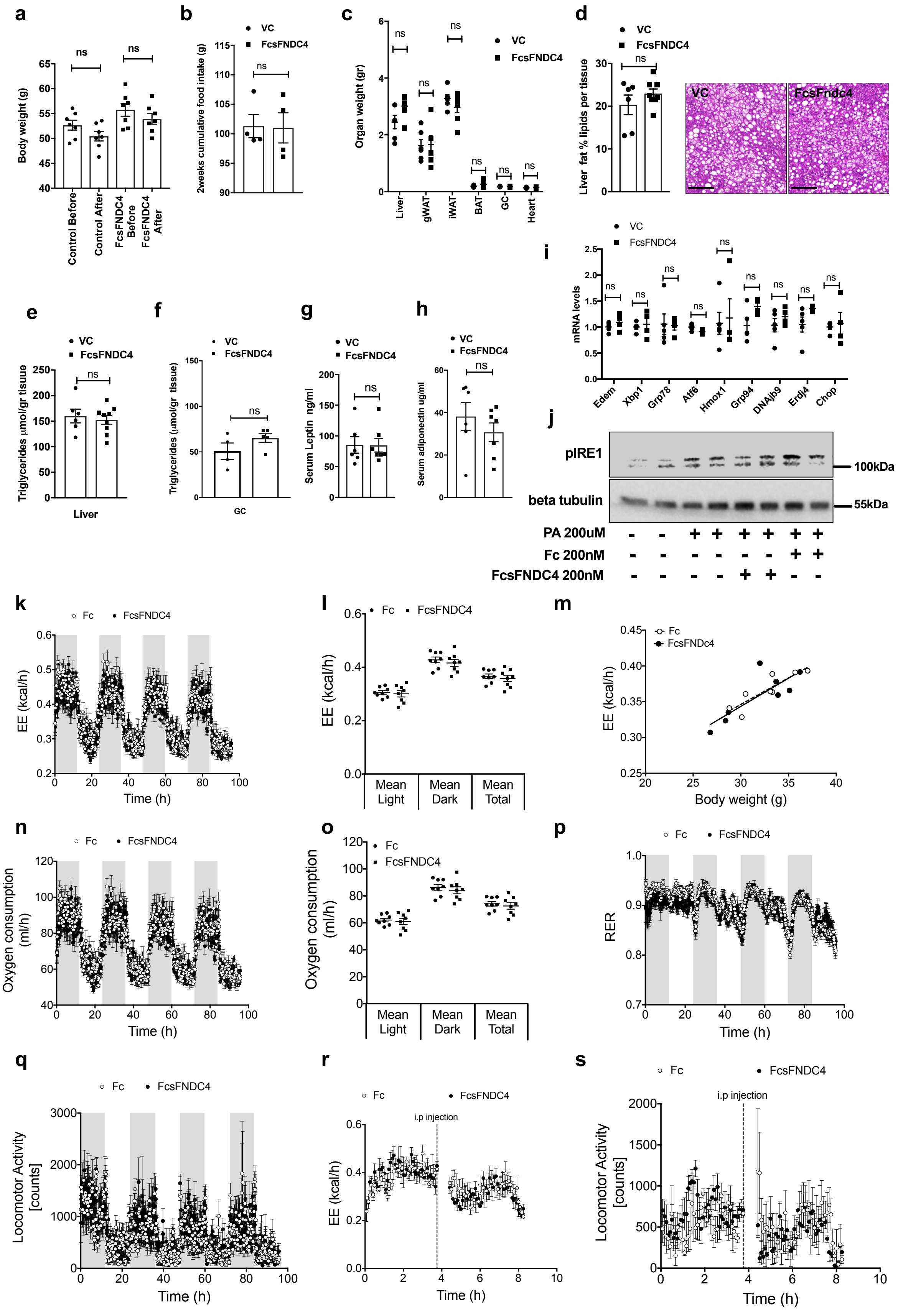
a
b
c
d
e
f
g
h
i
j
k
l
m
n
o
p
q
r
s
VC
FcsFNDC4
ns
Body weight (g)
Control Before
Control After
FcsFNDC4 Before
FcsFNDC4 After
2weeks cumulative food intake (g)
Organ weight (gr)
Liver
gWAT
iWAT
BAT
GC
Heart
Liver fat % lipids per tissue
FcsFndc4
Triglycerides μmol/gr tissue
Triglycerides (μmol/gr tissue)
Serum Leptin ng/ml
Serum adiponectin ug/ml
mRNA levels
Edem
Xbp1
Grp78
Atf6
Hmox1
Grp94
DNAjb9
Erdj4
Chop
pIRE1
beta tubulin
100kDa
55kDa
PA 200uM
Fc 200nM
FcsFNDC4 200nM
Fc
EE (kcal/h)
Time (h)
Mean Light
Mean Dark
Mean Total
FcsFNDc4
Body weight (g)
Oxygen consumption (ml/h)
RER
Locomotor Activity [counts]
i.p injection

Supplementary Fig.3 (related to Fig.3): Every second day injections of rec. FcsFNDC4 0.2 mg/kg improved glucose tolerance and increased glucose uptake in the white adipose tissue. **a** Body weight in grams before the start of injections and after 4 weeks of injections with FcsFNDC4 or vehicle control (VC). n=7 mice per group, per time point. **b** Cumulative food intake in grams for 2 weeks before the end of the study, n=4 cages, of 4 mice per cage, per group. **c** Organ weights at the end of the study, n=6 mice for VC and n=7 mice per group for FcsFNDC4. **d**-left panel: Quantification of % of lipid per total area of liver tissue observed by histology (n=6 mice for VC and n=8 mice for FcsFNDC4) and **d**-right panel: representative histology images out of 20 images per VC and FcFndc4 treated (4wks injections) HFD mice. HE. Bars = 200 μm. Triglyceride content in (**e**) liver (n=6 mice for VC and n=9 mice for FcsFNDC4) and (**f**) GC (n=4 mice for VC and n=5 mice for FcsFNDC4), **g** ELISA quantification of serum leptin (ng/ml) (n=6 mice for VC and n=7 mice for FcsFNDC4) and **h** serum adiponectin (μg/ml) (n=6 mice for VC and n=7 mice for FcsFNDC4) in VC and FcsFNDC4 treated (4 wks injections) HFD mice. **i** RT-qPCR quantification of indicated genes in gWAT (gonadal WAT) of HFD mice treated with FcsFNDC4 or Fc control (4wks injections) (n=5 mice for VC and n=4 mice for FcsFNDC4). Data shown are 2^ddCt values and *Hprt* is used as a housekeeping gene. **j** WB of pIRE1 and beta tubulin loading control on 3T3L1 lysates treated Palmitate (PA) 200uM in the presence or absence of FcsFNDC4 (200nM) and Fc (200nM) control for 24hr. Representative experiment out of 2 experiments is shown. Uncropped blots are available in the SourceData file. (**k-s**) Metabolic phenotyping (TSE) of HFD mice (16 weeks on HFD 45% fat) injected for 4 weeks with FcsFNDC4 or Fc control (0,2 mg/kg): Energy expenditure **k** longitudinal, **l** mean, **m** in correlation to body weight. Oxygen consumption **n** longitudinal, **o** mean. **p** Respiratory exchange ratio (RER), **q** locomotor activity. **k-q** n=8 mice per group. (**r,s**) Metabolic phenotyping of chow fed, lean young mice (12-14 weeks old) 4h prior and 2h post single injection of Fc or FcsFNDC4, 1 mg/kg, under fasting conditions: **r** Energy expenditure (EE) and **s** locomotor activity. In **r, s** n=4 mice per group. Mice are C57BL6N males. Bars shown are mean $\pm$ SEM. For **a-i** Statistics represent unpaired two-tailed t test. ns; non-significant. Source data are provided as a Source Data file.

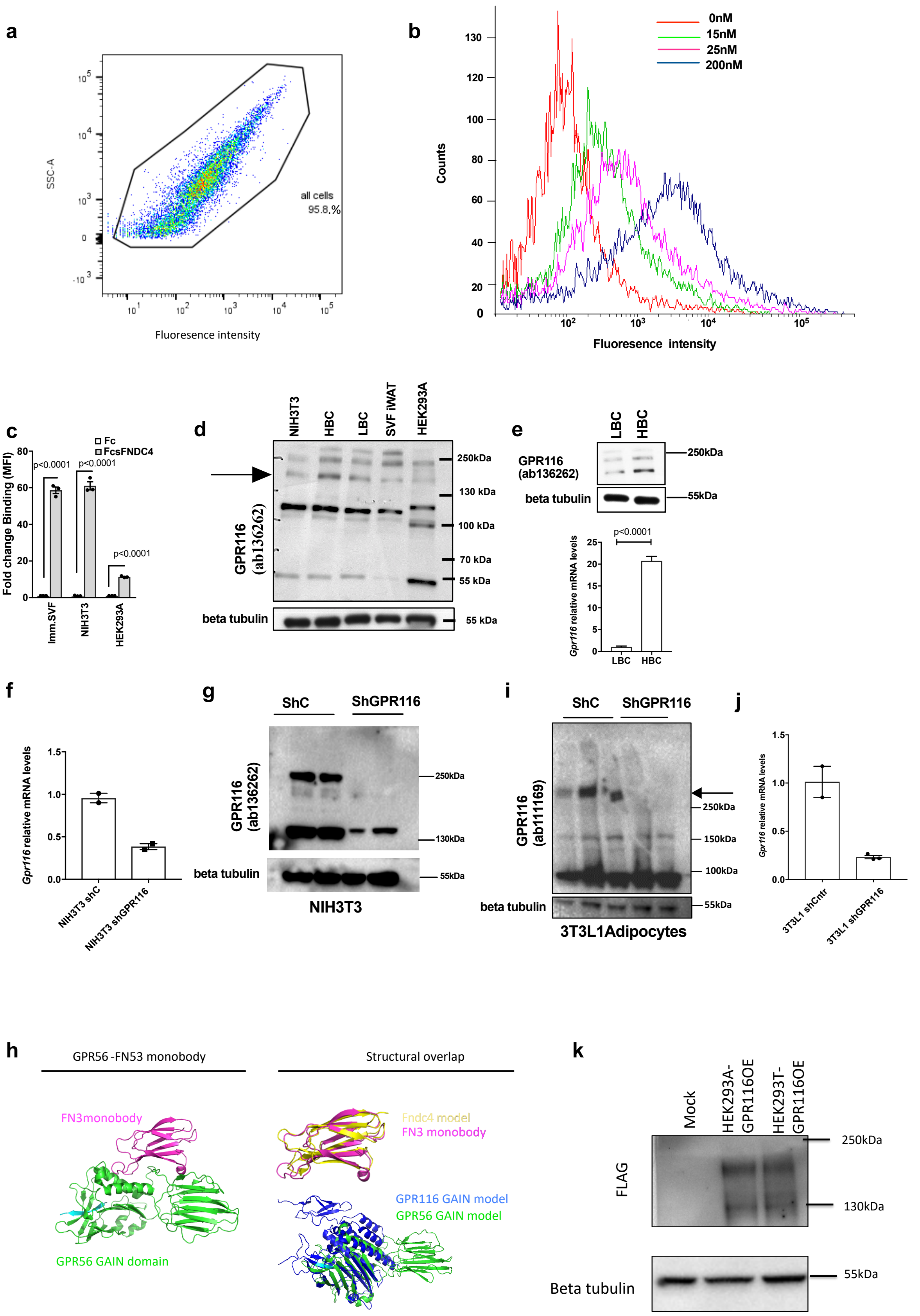

a
SSC-A
all cells
95.8%
Fluoresence intensity
b
0nM
15nM
25nM
200nM
Counts
Fluoresence intensity
c
Fc
FcsFNDC4
Fold change Binding (MFI)
p<0.0001
Imm.SVF
NIH3T3
HEK293A
d
NIH3T3
HBC
LBC
SVF iWAT
HEK293A
GPR116 (ab136262)
250kDa
130 kDa
100 kDa
70 kDa
55 kDa
beta tubulin
e
LBC
HBC
GPR116 (ab136262)
beta tubulin
250kDa
55kDa
Gpr116 relative mRNA levels
p<0.0001
f
Gpr116 relative mRNA levels
NIH3T3 shC
NIH3T3 shGPR116
g
ShC
ShGPR116
GPR116 (ab136262)
250kDa
130kDa
beta tubulin
55kDa
NIH3T3
i
ShC
ShGPR116
GPR116 (ab111169)
250kDa
150kDa
100kDa
beta tubulin
55kDa
3T3L1Adipocytes
j
Gpr116 relative mRNA levels
3T3L1 shCntr
3T3L1 shGPR116
h
GPR56 -FN53 monobody
Structural overlap
FN3monobody
GPR56 GAIN domain
Fndc4 model
FN3 monobody
GPR116 GAIN model
GPR56 GAIN model
k
Mock
HEK293A-GPR116OE
HEK293T-GPR116OE
FLAG
250kDa
130kDa
Beta tubulin
55kDa

Supplementary Fig.4 (related to Fig.4): a Representative gating strategy for all FACS experiments presented in Figure 4-panels: **a,b,f,h,i,k,m,n**. The whole population of ells (approx. 95% of all cells) was selected. **b** Histogram: Typical shift in fluorescence, demonstrating increasing fluorescence intensity (FI), upon increasing binding of rec. FcsFNDC4 on assessed cells (all cells-total population was selected at gating (see **a**). **c** Binding (MFI) of FcsFNDC4 and Fc proteins at 100 nM, to various cell lines. Values correspond to fold change of MFI (per 10000 cells/events) normalized to the MFI of Fc binding (Set at 1). n=3 replicate wells per group. This experiment was performed at least 3 times. **d** Western blot analysis of GPR116 protein at indicated cell types. Beta tubulin was used as a loading control. Arrow shows GPR116 band corresponding to the one identified by pull down in Fig.4j. This WB was preformed once. **e** WB anti-GPR116 against endogenous GPR116 (top panel) and q-PCR measured *Gpr116* mRNA in HBC and LBC (bottom panel). This image was cropped and reversed from WB shown in **d** and it is repeated here for clarity. **f** q-PCR quantification of *Gpr116* mRNA in NIH3T3 preadipocytes (n=2 replicate wells of one experiment). **g** WB against GPR116 in total cells lysates of NIH3T3 preadipocytes. This experiment was performed once. **h** Interaction model of the GPR56-GAIN domain and FN3 monobody crystal structure, pdb 5KVM (https://www.rcsb.org/structure/5KVM). Predicted structural overlap of the FNDC4-FN3 domain and GPR116-GAIN domain with the corresponding GPR56-GAIN and FN3 monobody. Both FNDC4-FN3 and GPR116-GAIN domain models were originated based of the first hit of the HHpred [3] using the primary sequence of each protein. WB against GPR116 (**i**) and mRNA levels of GPR116 (**j**) in 3T3L1 mature adipocytes transduced with lentivirus shGRP116 (mouse) and shControl. n=2 for ShControl and n=3 for shGPR116 replicate of a representative experiment out of three experiments. **k** WB against FLAG on total cell lysates of HEK293A and HEK293T cells stably overexpressing human GPR116-Cterm.FLAG or mock control. This WB experiment was performed once. For **e** (bottom panel), **f, j** data shown are 2^ddCt values and *Tbp* was used as a housekeeping gene. In **d**, **e**, **g** Anti-GPR116 used: ab136262. In **i**, **j** we used anti-GPR116 ab111169. Bars are mean $\pm$ SEM. For **c, e (bottom)** Statistics represent unpaired two tailed t test. Source data are provided as a Source Data file.

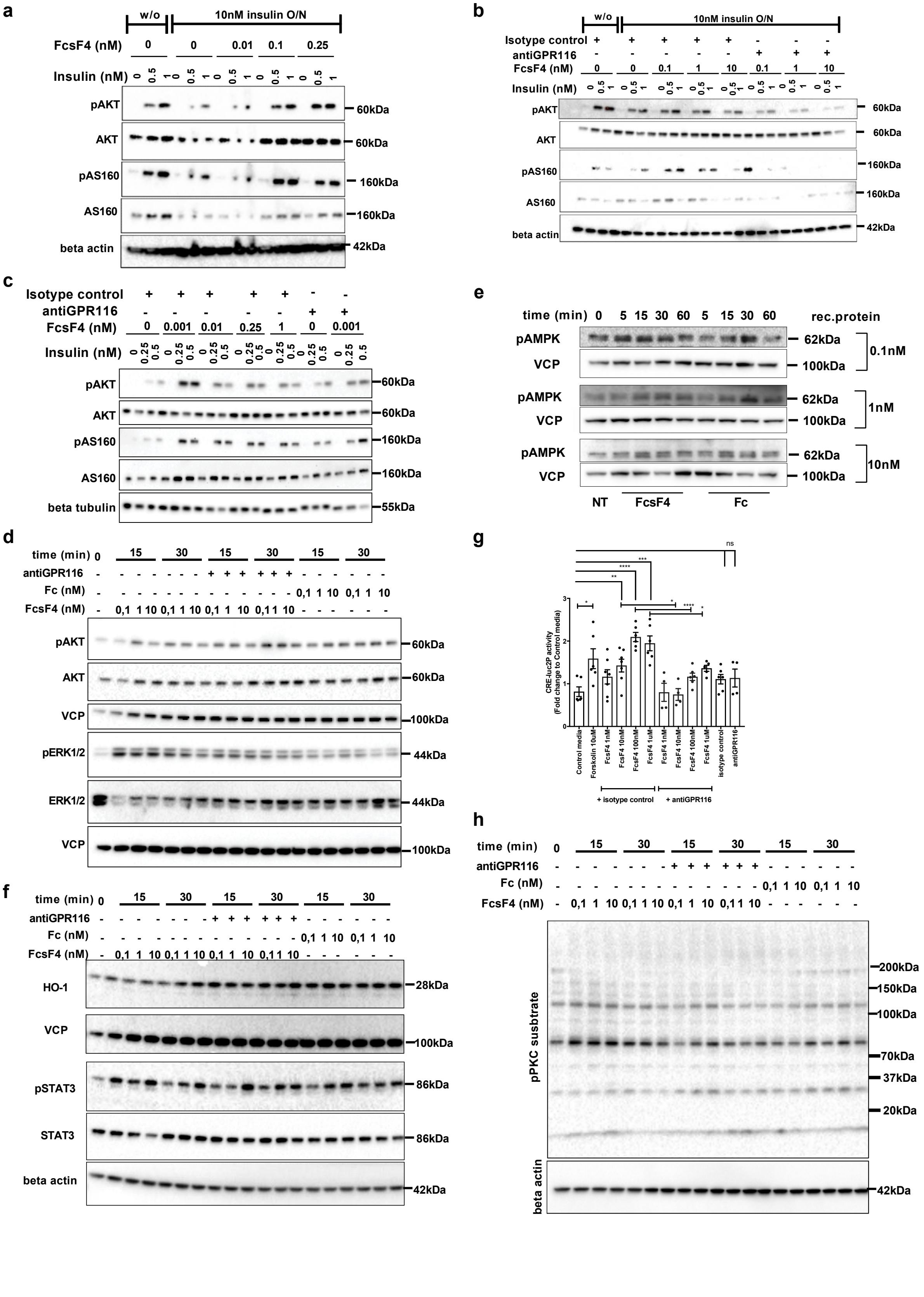
a
w/o
10nM insulin O/N
FcsF4 (nM) 0 0 0.01 0.1 0.25
Insulin (nM)
pAKT
AKT
pAS160
AS160
beta actin
60kDa
160kDa
42kDa
b
w/o
10nM insulin O/N
Isotype control
antiGPR116
FcsF4 (nM)
Insulin (nM)
pAKT
AKT
pAS160
AS160
beta actin
60kDa
160kDa
42kDa
c
Isotype control
antiGPR116
FcsF4 (nM) 0 0.001 0.01 0.25 1 0 0.001
Insulin (nM)
pAKT
AKT
pAS160
AS160
beta tubulin
60kDa
160kDa
55kDa
d
time (min) 0 15 30 15 30 15 30
antiGPR116
Fc (nM)
FcsF4 (nM)
pAKT
AKT
VCP
pERK1/2
ERK1/2
VCP
60kDa
100kDa
44kDa
e
time (min) 0 5 15 30 60 5 15 30 60
rec.protein
pAMPK
VCP
62kDa
100kDa
0.1nM
1nM
10nM
NT
FcsF4
Fc
f
time (min) 0 15 30 15 30 15 30
antiGPR116
Fc (nM)
FcsF4 (nM)
HO-1
VCP
pSTAT3
STAT3
beta actin
28kDa
100kDa
86kDa
42kDa
g
CRE-luc2P activity (Fold change to Control media)
Control media
Forskolin 10uM
FcsF4 1nM
FcsF4 10nM
FcsF4 100nM
FcsF4 1uM
isotype control
antiGPR116
+ isotype control
+ antiGPR116
ns
h
time (min) 0 15 30 15 30 15 30
antiGPR116
Fc (nM)
FcsF4 (nM)
pPKC susbstrate
beta actin
200kDa
150kDa
100kDa
70kDa
37kDa
20kDa
42kDa

Supplementary Fig.5 (related to Fig. 6): FNDC4-GPR116 interraction promotes insulin sensitivity in 3T3L1 (mature) adipocytes. WB of indicated proteins (from total cell lysates) of 3T3L1 adipocytes treated with rec. FcsFDCN4 (FcsF4) at indicated concentrations and insulin 10nM O/N (**a**) in the absence or presence of 0,4ug/ml anti-GPR116 (ab111169) blocking antibody or 0,4ug/ml isotype control (**b**). Cells were serum starved in DMEM high glucose for 3 h, followed by 5 minutes insulin stimulation at indicated concentrations. **a,b** are representative experiments out of at least two independent experimensts. Experiments repeats are also shown in **Figure 6a,b**. **c** WB of indicated proteins (from total cell lysates) of 3T3L1 adipocytes treated 5min with insulin plus FcsFNDC4 at indicated concenctrations, 3h after serum starvation. For indicated conditions, 30min before the inuslin stimulation plus FcsFNDC4, 0.4ug/ml anti-GPR116 (ab111169) or isotype control were added to the cells. Representative results out of at least two independent experiments are shown. **d,f,h** WB of indicated proteins (from total cell lysates) of mouse primary SVF derived adipocytes. Adipocytes were serum starved for 3h prior the stimulation with FcsFNDC4 or Fc at indicated conenctrations (in the absence of insulin) at indicated times points. For indicated conditions, 30min prior the stimulation with rec. protein the adipocytes were treated with 0,4ug/ml anti-GPR116 (ab111169). **e** WB of indicated proteins (from total cell lysates) of SVF derived mouse adipocytes treated at indicated time points with rec. protein Fc or FcsFNDC4 (0,1nM, 1nM, 10nM). At **d,e,f,h** representative results out of two independent experiments are shown. **g** Stimulation (3-4h) of 3T3L1 adipocytes CRE-luciferase reporter stable cells lines with indicated concentrations of rec. protein or Forskolin. For indicated conditions antiGPR116 (ab111169) 0.4ug/ml was added 30 min prior the addition of the rec. proteins. Data point represent replicate wells per condition. This experiment was performed once under the exact same conditions. Four independent explerimental repeats of all conditions except of the FcsF4 1uM are shown in Figure 6e. At **g** Statistics represent unpaired two tailed t test and exact p values are for the comparison control-Fosrkolin p=0.0136, Control-FcsF410nM p=0.0089, Control – FcsF4100nM p<0.0001, Control-FcsF41uM p=0.0003. FcsF410uM isotype control-FcsF410nMantiGpr116 p=0.014, FcsF4100nM isotype control –FcsF4 10nM antiGPR116 p<0.0001, FcsF41uM isotype control- FcsF41uM antiGPR116 p=0.017. Phospho-antibodies :pAKT^{Ser473}, pAMPK^{Thr172}, pSTA3^{Y705}, pCREB^{Ser133}. Source data are provided as a Source Data file.

a (quantification of WB Fig.6a)

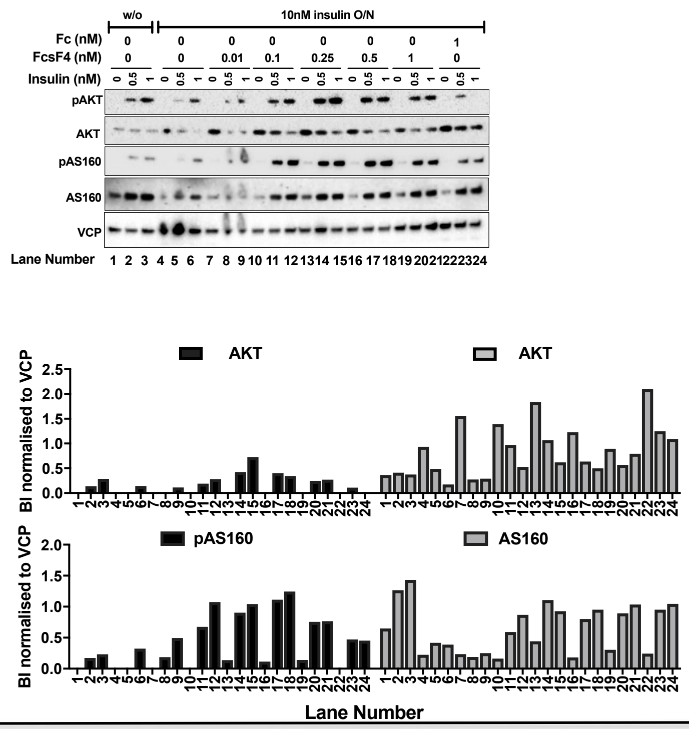

	pAKT	AKT	pAS160	AS160	VCP
1	0	185270	0	330777	510096
2	68890	209850	88380	645507	370212
3	146090	190350	119110	730935	533628
4	0	477440	0	114165	964032
5	0	249630	0	213030	1869204
6	73520	88000	164380	197604	946632
7	0	794660	0	119178	490248
8	0	140210	95640	97146	272797
9	57070	148680	253130	127575	244878
10	0	708760	0	82818	327872
11	96710	495900	344930	301716	372010
12	142890	269790	548660	442404	459774
13	0	936520	69860	224910	491660
14	217350	543360	460530	564588	683224
15	371160	315850	532150	472572	387268
16	0	625830	58220	92259	465790
17	204570	323700	566940	408771	411968
18	175690	254030	634260	485712	383552
19	0	455640	72300	155448	526224
20	125540	290280	384890	455274	639840
21	137210	405150	390620	528183	916096
22	0	1069870	0	123372	693336
23	55260	635650	240420	485478	656820
24	0	557840	231390	533682	539232

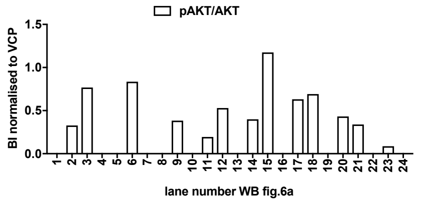

b (quantification of WB Fig.6b)

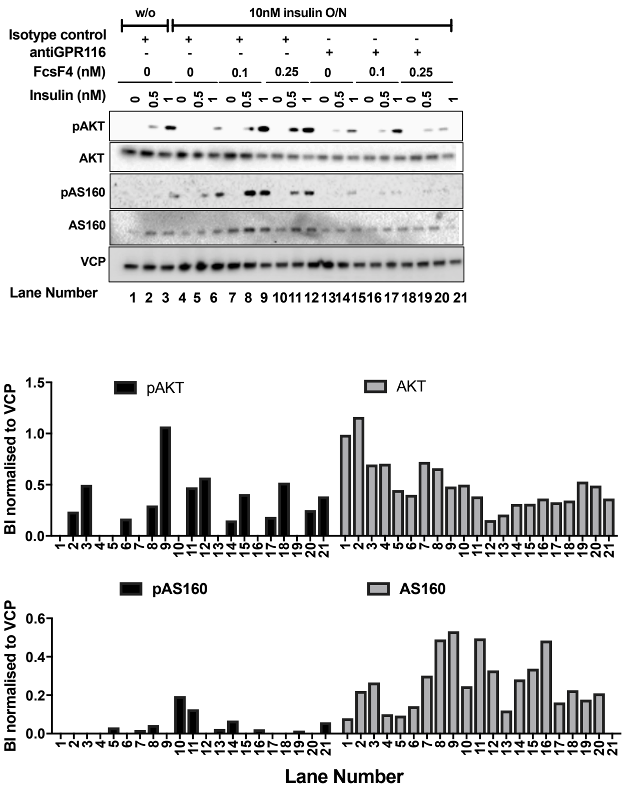

	pAKT	AKT	pAS160	AS160	VCP
1	0	1167133	0	94959	1181850
2	299688	1466740	0	279792	1262268
3	579320	809379	0	309429	1161090
4	0	1107900	0	158328	1571823
5	0	786438	57340	165087	1758879
6	305872	724239	0	257832	1804311
7	0	1190709	30770	497214	1647621
8	401296	893250	59940	661968	1351638
9	914312	411831	0	455373	855180
10	0	493335	192360	243198	985086
11	505464	411867	135320	528750	1066518
12	870952	235386	0	502920	1530450
13	0	365463	43180	211284	1749456
14	176528	363726	79400	328428	1162305
15	378552	289710	0	312390	925632
16	0	339183	21080	450234	929898
17	195560	346689	0	170568	1053540
18	517216	343440	0	224910	994086
19	0	582057	17390	194418	1097847
20	256128	497475	0	212571	1013643
21	281632	266013	43330	0	729972

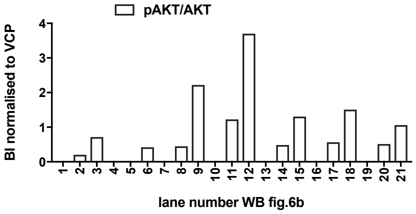

Supplementary Fig. 6 (related to Fig. 6) : Quantification of WB shown in Fig. 6. Image Lab was used to calculate band intensities (BI).

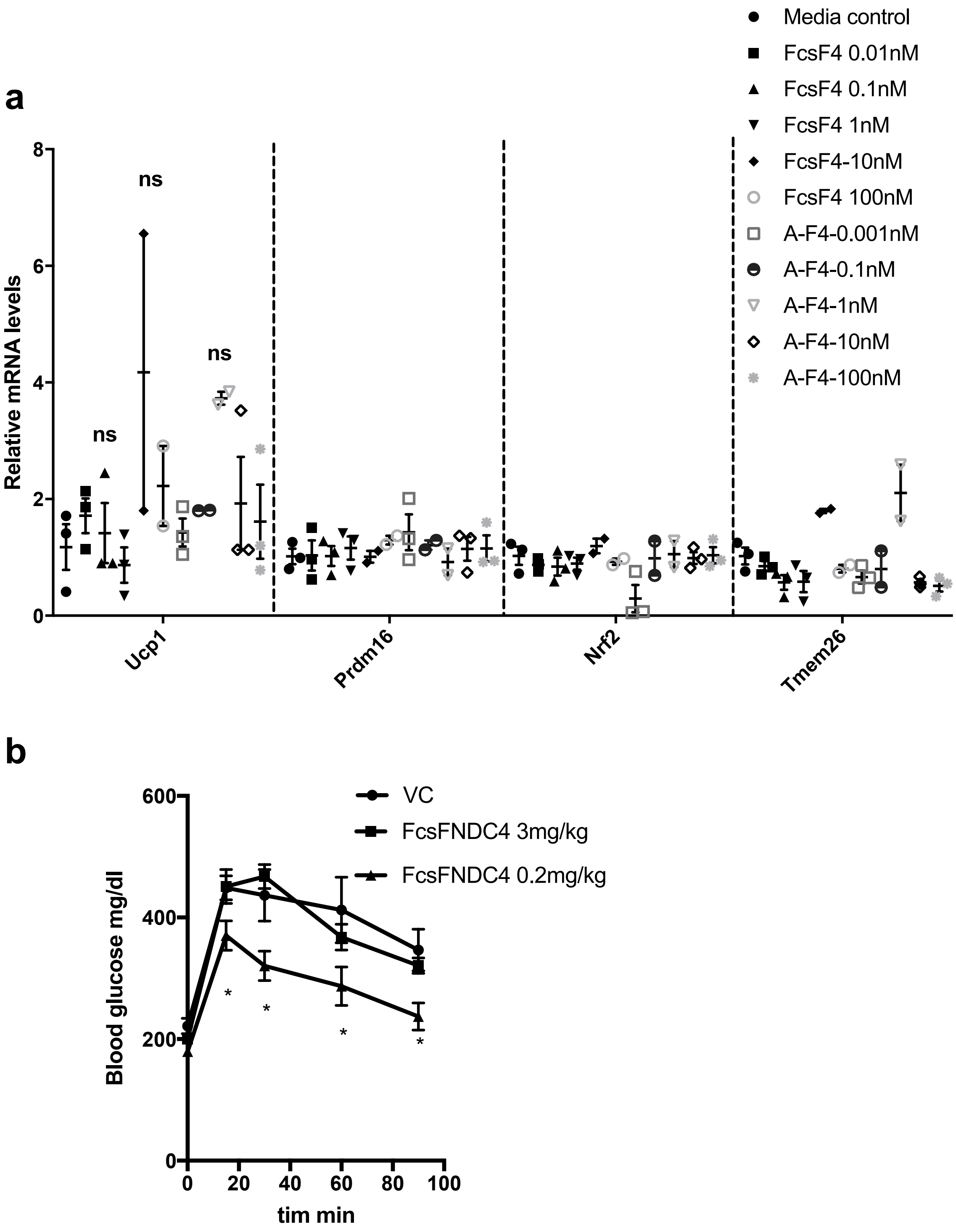

Supplementary Fig. 7 (related to data mentioned at the Discussion) . a Mouse primary SVF dervived matured adipocytes treated 24h with different concentrations of mammalian FcsFNDC4

(used in this paper) or e.coli derived native sFNDC4 from Adipogen. Indicated browning and mitochondrial genes were quantified by q-PCR. mRNA levels represent 2^ddCt values relative to the control media. This experiment was performed at least 3 two times (higher concentration 100nM were repeated only in independent experiments). n= 2-3 replicate wells of a representative experiment. This exact experiment with all presented concentrations of rec. protein assessed in parallel was performed only once. **b** Blood glucose levels duting an IPGTT challenge on HFD (60% fat) mice, which had been injected for 4 weeks with VC (vehicle control), FcsFNDC4 3mg/kg or FcsFNDC4 0,2mg/kg (every second day injections). n=6 mice per group. In **b** Statistics represent unpaired two tailed t test comparing VC and FcsFNDC4 0.2mg/kg and * indicates $p<0.05$ for indicated time points during the IPGTT. Source data are provided as a Source Data file.

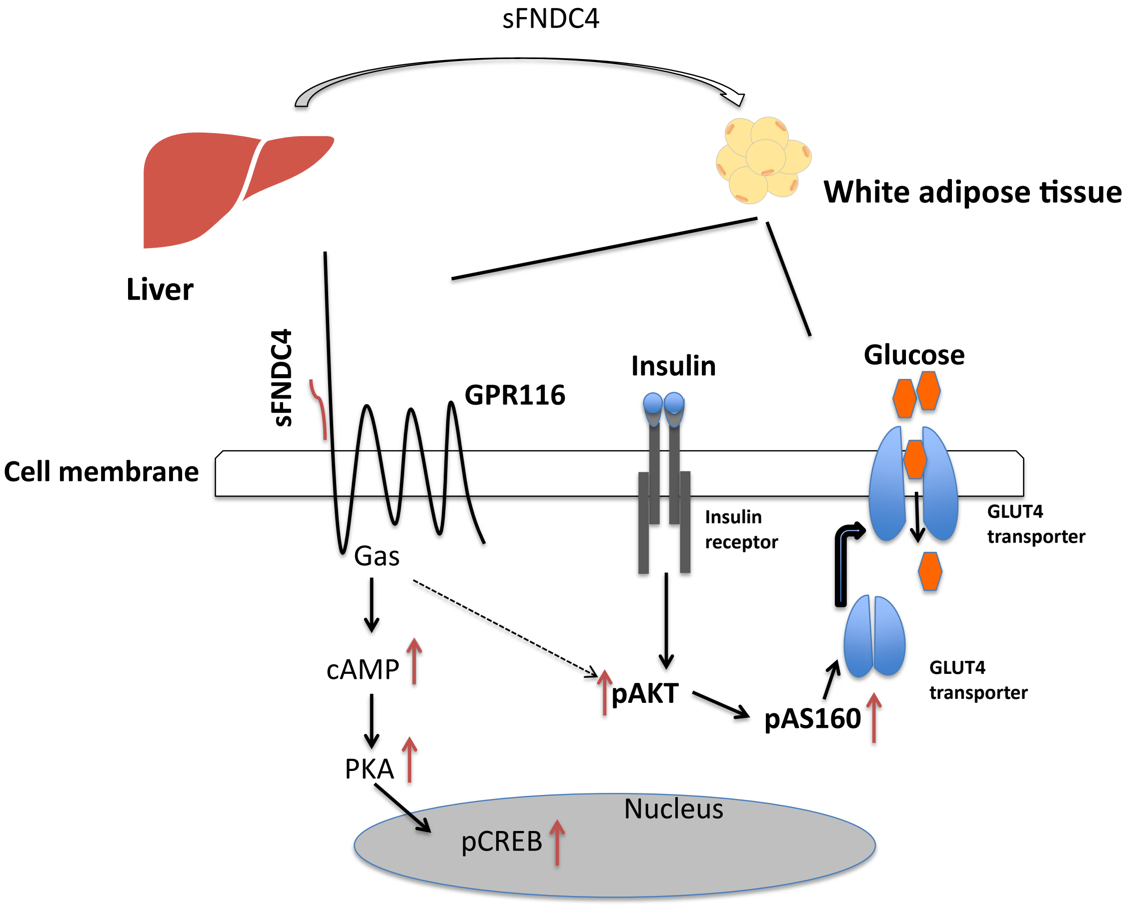

Supplementary Fig.8: Model of the endocrine effects of the hepatokine sFNDC4. sFNDC4 circulating levels are regulated by the liver. sFNDC4 is secreted by the liver and enchances insulin signaling in the WAT via bidning to the GPR116. Binding of sFNDC4 to GPR116 stimulates Gs-cAMP- PKA signaling which leads to phosphorylation to pCREB. These events prime insulin receptor signaling and upon insulin stimulation crosstalk with the pAKT-pAS160-GLUT4 pathway to promote glucose uptake in WAT. Abbreviations: GPR116, G-protein coupled receptor 116, sFNDC4, soluble Fibronectin Type III Domain Containing 4, GLUT4 Glucose transporter type 4, pCREB, phospho cAMP-response element-binding protein, cAMP, Cyclic adenosine monophosphate. PKA, protein kinase A, pAKT, phospho protein kinase B. Thick black arrow lines indicate signaling events which were elucidated experimentally in the present manuscript. Black dashed arrow line indicates a crosstalk or interaction, which can be

inferred from the data presented in the present manuscript, but still requires further experimental validation. Red upwards arrow thick lines indicate upregulation.

Supplementary Tables:

For Supplementary Table 1 and Table 2, Source data are provided as a Source Data file.

Supplementary Table 1 (related to Figure 1): Human cross sectional cohort Leipzig. Table of mean and SEM values of study grou

Group		Age (yrs)	BMI (kg/m²)	WHR	Body fat (%)	FPG (mmol/l)	FPI (pmol/l)	2 h OGTT (mmol/l)	Clamp GIR (µmol/kg/min)	HbA1c (%)	Total Cholesterol (mg/dl)	HDL-Cholesterol (mg/dl)	LDL-Cholesterol (mg/dl)	TG (mg/dl)	FFA (mmol/l)
Lean-ND	Number of values	12	12	12	12	12	12	12	12	12	12	12	12	12	12
	Mean	64.33	24.53	0.8925	22.43	5.26	16.78	5.971	101.2	5.275	171.4	53.56	106.1	79.93	0.3758
	Std. Error of Mean	4.51	0.257	0.02097	0.5371	0.09957	3.463	0.1982	3.289	0.05094	7.172	4.298	10.23	10.24	0.06258
Obese-IGT/IIT	Number of values	28	28	28	28	28	28	28	28	28	28	28	28	28	28
	Mean	79.04	34.2	1.063	36.73	5.246	126.1	6.105	59	5.593	195.6	51.84	106	159.3	0.5229
	Std. Error of Mean	4.17	1.11	0.0268	1.526	0.09411	15.26	0.1461	4.522	0.04598	5.624	1.961	5.158	10.6	0.0525
Obese-T2D	Number of values	10	10	10	10	10	10	3	10	10	10	10	10	10	10
	Mean	69	34.62	1.048	33.81	6.281	318.8	13.73	30.8	6.04	228.8	52.5	135.9	218.2	0.718
	Std. Error of Mean	4.712	1.584	0.03323	2.772	0.2263	39.7	1.426	6.178	0.1688	7.183	1.905	7.149	12.34	0.1052
	p<0.05	a	a,b	a,b	a,b	a,b,c	a,b,c	b,c	a,b,c	a,b,c	a,b,c	ns	b,c	a,b,c	b,c

Lean-ND:Lean- no diabetes, Obese-IGT/IIT: impaired glucose tolerance/ impaired insulin tolerance , Obese-T2D: Obese type 2 diabetes
Comparisons: a LeanND-Obese-IGT/IIT, b LeanND--Obese-IGT/IIT, c Obese-IGT/IIT-ObeseT2D
yrs:Years, BMI: Body mass index, WHR: waist to hip ratio, FPG: fasting plasma glucose, FPI: fasting plasmsa insulin, HbA1c: Hemoglobin A1c, HDL:High density lipoprotein, LDL: Low density liporotein, TG: triglycerides, FFA: free fatty acids

Supplementary Table 2 : Related to Figure 7a-g. Subjects information and metabolic parameters before and 12 months after the interventions :Bariatric surgery, Diet/exercise

Intervention	Gender	Age		Diabetes		Height (cm)	Body weight (kg)		BMI (kg/m²)		Fasting plasma glucose (mmol/l)		HbA1c (%)		Fasting plasma insulin (pmol/l)		C-Peptid (nmol/l)		gGT (µkat/l)		CRP (mg/l)		Triglycerides (mmol/l)		FFA (mmol/l)		Cholesterol total (mmol/l)		HDL-Cholesterol (mmol/l)		LDL-Cholesterol (mmol/l)		sFNDC4 ng/ml	
	Baseline	Baseline	12 months	Baseline	12 months	Baseline	Baseline	12 months	Baseline	12 months	Baseline	12 months	Baseline	12 months	Baseline	12 months	Baseline	12 months	Baseline	12 months	Baseline	12 months	Baseline	12 months	Baseline	12 months	Baseline	12 months	Baseline	12 months	Baseline	12 months	Baseline	12 months
diet/exercise	M	46	47	T2D	no	180	108	96	33.3	29.6	7.18	4.94	7	6.1	84.5	92.3	0.52	0.656	0.31	0.34	8.82	2.38	1.71	1.63	0.38	0.24	4.38	4.21	1.01	1.27	2.74	2.85	55.52	57.46
diet/exercise	M	56	57	T2D	no	178	96	32	30.3	10.1	5.78	5.79	5.3	5.3	46.8	49.2	0.7	0.56	0.76	0.69	8.84	0.37	0.78	0.77	0.32	0.45	5.62	5.81	1.47	1.32	3.85	4.03	49.18	116.79
diet/exercise	M	50	51	T2D	no	168	117	111	41.5	39.3	8.72	4.48	6.1	5.7	207.5	54.6	2.5	0.85	1.55	1.24	6.51	3.58	4.11	3.52	0.34	0.45	7.07	6.52	1.06	1.54	4.58	4.67	51.21	58.81
diet/exercise	M	48	49	T2D	no	174	112	113	37.0	37.3	5.85	5.81	6.4	5.6	125.2	67.6	1.24	0.73	0.58	0.54	3.33	1.22	2.07	2.11	1.25	1.09	5.04	4.73	1.28	1.33	3.01	2.74	48.38	48.02
diet/exercise	M	54	55	T2D	no	180	113	110	34.9	34.0	8.17	5.31	7.9	5.9	297.4	285	2.66	2.55	1.99	2.42	6.97	3.74	1.24	1.02	0.23	0.21	4.67	4.76	1.11	1.29	3.12	2.89	47.18	51.28
Mean		50.8	51.8			176	109.20	92.40	35.39	30.07	7.14	5.27	6.54	5.72	152.28	109.74	1.52	1.07	1.04	1.05	6.89	2.26	1.98	1.81	0.50	0.49	5.36	5.21	1.19	1.35	3.46	3.44	50.29	66.47
Student´s T-test p-value							0.2338		0.2315		0.0856		0.0708		0.2278		0.2236		0.9501		0.0185		0.2036		0.8046		0.3257		0.1937		0.8104		0.2789	

Intervention	Gender	Age		Diabetes		Height (cm)	Body weight (kg)		BMI (kg/m²)		Fasting plasma glucose (mmol/l)		HbA1c (%)		Fasting plasma insulin (pmol/l)		C-Peptid (nmol/l)		gGT (µkat/l)		CRP (mg/l)		Triglycerides (mmol/l)		FFA (mmol/l)		Cholesterol total (mmol/l)		HDL-Cholesterol (mmol/l)		LDL-Cholesterol (mmol/l)		sFNDC4 ng/ml	
	Baseline	Baseline	12 months	Baseline	12 months	Baseline	Baseline	12 months	Baseline	12 months	Baseline	12 months	Baseline	12 months	Baseline	12 months	Baseline	12 months	Baseline	12 months	Baseline	12 months	Baseline	12 months	Baseline	12 months	Baseline	12 months	Baseline	12 months	Baseline	12 months	Baseline	12 months
diet/exercise	F	52	53	T2D	no	159	101	95	40.0	37.6	9.16	5.49	6.7	5.9	344.6	46.9	2.15	0.62	0.44	0.49	5.49	1.82	2.06	2.18	0.28	0.2	5.43	5.32	1	1.2	3.71	3.95	48.6	54.5
diet/exercise	F	54	55	T2D	no	168	97	85	34.4	30.1	9.37	4.76	6.9	5.8	30.5	19.9	0.65	0.38	0.45	0.31	2.24	1.33	1.68	1.65	0.46	0.41	5.12	5.06	1.13	1.45	3.42	3.64	52.0	55.3
diet/exercise	F	53	54	T2D	no	173	95	84	31.7	28.1	6.9	5.71	7.6	5.9	117.0	88.7	0.56	0.85	0.79	0.42	3.95	2.56	3.32	2.98	0.21	0.15	6.39	6.11	1	0.96	4.43	4.66	61.1	65.8
diet/exercise	F	58	59	T2D	no	158	78	72	31.2	28.8	5.46	4.73	6.2	5.9	124.3	99.5	0.6	1.03	0.64	0.67	6.23	2.61	1.4	1.52	0.38	0.26	4.04	4.23	1.5	1.67	2.14	2.16	58.4	64.4
diet/exercise	F	52	53	T2D	no	166	69	72	25.0	26.1	6.92	4.98	6.5	5.9	521.6	181.3	2.89	2.15	1.08	1.22	8.26	8.75	2.93	3.06	0.64	0.39	6.79	6.8	1.01	1.29	4.63	4.5	46.2	46.6
diet/exercise	F	49	50	T2D	no	179	93	85	29.0	26.5	7.1	5.10	6.1	5.8	133.4	38.7	0.93	0.76	0.28	0.22	1.53	1.04	1.62	1.58	0.82	0.71	4.94	5.16	1.07	1.25	3.25	3.22	51.9	63.0
diet/exercise	F	56	57	T2D	no	158	75	72	30.0	28.8	6.47	5.43	6.1	5.8	133.6	92.4	1.27	1.88	0.6	0.63	7.9	8.24	2.34	1.63	0.79	0.74	5.63	5.54	0.97	1.24	3.79	2.56	50.6	59.8
diet/exercise	F	54	55	T2D	no	186	108	93	31.2	26.9	4.77	5.06	6.3	5.9	137.7	91.0	1.52	1.06	1.71	1.55	5.06	3.94	1.52	1.41	0.46	0.3	6.93	7.21	1.4	1.42	4.66	4.91	50.6	58.1
diet/exercise	F	53	54	T2D	no	165	92	84	33.8	30.9	7.25	4.63	6.8	6.1	175.5	44.8	2.38	0.67	1.38	1.45	0.41	9.2	1.28	1.12	0.24	0.21	6.83	7.05	1.48	1.5	4.6	4.45	49.7	54.8
diet/exercise	F	52	53	T2D	no	170	97	92	33.6	31.8	6.95	7.55	8.2	5.9	123.8	31.9	1.92	0.917	4.56	5.96	3.09	4.13	1.8	1.81	0.38	0.33	4.36	4.5	1.24	1.63	2.44	2.59	52.9	56.2
diet/exercise	F	51	52	T2D	no	170	107	102	37.0	35.3	7.12	5.81	6.1	5.6	142.6	46.2	2.62	0.63	1.34	0.83	3.07	9.4	1.86	1.22	0.41	0.42	5.24	5.03	1.05	1.42	3.4	3.25	55.2	60.0
diet/exercise	F	54	55	T2D	no	173	114	109	38.1	36.4	10.86	5.19	7.1	6	370.9	200.6	0.59	1.82	0.42	0.32	2.91	6.16	2.34	1.92	0.49	0.55	5.68	5.66	1.34	1.39	3.22	3.26	50.6	58.8
diet/exercise	F	56	57	T2D	no	164	113	105	42.0	39.0	4.77	5.41	7.5	5.9	36.5	26.1	0.73	0.52	1.08	1.24	0.67	3.28	1.5	1.63	0.38	0.36	5.31	5.18	1.33	1.82	3.37	3.81	50.9	56.1
diet/exercise	F	53	54	T2D	no	153	73	69	31.2	29.5	6.57	5.32	6.3	5.8	167.8	179.7	2.29	1.91	0.68	0.62	4.66	7.47	3.24	4.05	0.82	0.82	8.37	7.6	1.22	1.45	5.66	5.79	41.7	55.7
diet/exercise	F	51	52	T2D	no	167	99	100	35.5	35.9	9.56	5.61	7.9	6.1	99.1	55.2	2.4	N/A	1.72	1.26	6.52	0.96	2.58	2.82	0.92	0.83	3.9	3.84	0.83	1.05	2.31	2.22	55.8	61.6
diet/exercise	F	50	51	T2D	no	170	87	95	30.1	32.9	5.24	4.75	6.5	5.7	54.5	31.8	0.48	0.39	1.09	0.83	5.88	9.76	1.14	1.06	1.06	1.24	4.96	5.22	1.27	1.3	3.29	3.39	51.7	54.3
diet/exercise	F	48	49	T2D	no	158	78	79	31.2	31.6	3.9	5.13	6.8	5.9	61.9	20.8	0.35	0.33	1	0.82	14.38	1.98	1.71	1.43	0.52	0.24	6.32	5.98	1.18	1.34	4.71	4.7	58.4	59.3
diet/exercise	F	58	59	T2D	no	150	88	86	39.1	38.2	8.77	6.00	6.9	5.7	308.3	165.1	2.69	1.06	0.78	0.72	8.01	0.92	2.66	3.18	0.71	0.56	4.77	4.82	0.79	0.83	3.09	2.88	46.5	53.7
diet/exercise	F	45	46	T2D	no	162	127	125	48.4	47.6	8.11	5.62	6.5	5.8	199.8	90.5	1.67	1.1	0.41	0.44	2.89	3.18	1.94	1.75	0.65	0.29	5.30	5.4	1.52	1.69	2.87	3.02	46.8	53.7
Mean		52.6	53.6			165.7	94.3	89.7	34.4	32.7	7.1	5.4	6.8	5.9	172.8	81.6	1.5	1.0	1.1	1.1	4.9	4.6	2.0	2.0	0.6	0.5	5.6	5.6	1.2	1.4	3.6	3.6	51.5	57.5
Student´s T-test p-value							0.0018		0.0012		0.0007		0.0000		0.0005		0.0386		0.7957		0.7647		0.5679		0.0076		0.5955		0.0000		0.9842		0.0000	

Intervention	Gender	Age		Diabetes		Height (cm)	Body weight (kg)		BMI (kg/m²)		Fasting plasma glucose (mmol/l)		HbA1c (%)		Fasting plasma insulin (pmol/l)		C-Peptid (nmol/l)		gGT (µkat/l)		CRP (mg/l)		Triglycerides (mmol/l)		FFA (mmol/l)		Cholesterol total (mmol/l)		HDL-Cholesterol (mmol/l)		LDL-Cholesterol (mmol/l)		sFNDC4 ng/ml	
	Baseline	Baseline	12 months	Baseline	12 months	Baseline	Baseline	12 months	Baseline	12 months	Baseline	12 months	Baseline	12 months	Baseline	12 months	Baseline	12 months	Baseline	12 months	Baseline	12 months	Baseline	12 months	Baseline	12 months	Baseline	12 months	Baseline	12 months	Baseline	12 months	Baseline	12 months
diet/exercise	M	44	45	T2D	T2D	167	90	88	32.3	31.6	6.44	6.03	6.4	6.5	73	95	0.97	1.87	2.53	3.06	3.34	4.28	1.37	1.32	0.75	0.91	4.31	4.33	1.7	1.95	2.04	1.92	45.8	47.9
diet/exercise	M	52	53	T2D	T2D	190	119	118	33.0	32.7	7.29	6.94	6.7	6.8	267	237	2.72	1.56	0.45	0.48	10.85	1.34	2.05	2.11	0.09	0.11	6.25	6.45	1.34	1.58	4.39	4.19	60.2	61.6
diet/exercise	M	57	58	T2D	T2D	180	85	88	26.2	27.2	10.9	5.94	8.6	8.8	135	132	2.32	1.36	2.05	1.84	9.56	0.69	1.4	1.42	1.28	0.92	4.32	4.18	1.25	1.22	2.64	2.37	67.0	59.3
diet/exercise	M	52	53	T2D	T2D	177	113	109	36.1	34.8	5.69	6.95	6.9	6.8	68	57	0.99	0.72	0.81	0.96	3.95	9.38	2.02	1.85	0.09	0.08	4.15	4.19	1.22	1.16	2.21	2.14	56.3	57.8
diet/exercise	M	54	55	T2D	T2D	178	114	106	36.0	33.5	15.14	8.63	8.8	7.9	143	155	1.15	1.24	0.34	0.32	9.14	2.23	1.35	1.39	0.22	0.26	3.7	3.81	1.21	1.08	1.98	2.31	56.7	56.5
diet/exercise	M	46	47	T2D	T2D	179	111	110	34.6	34.3	8.17	6.85	6.8	6.5	172	132	1.38	0.58	0.71	0.82	0.71	2.72	2.32	2.23	0.54	0.35	4.27	4.36	1.18	1.25	2.24	2.4	58.0	52.6
diet/exercise	M	52	53	T2D	T2D	183	135	132	40.3	39.4	6.47	6.33	6.5	6.3	271	153	2.02	1.88	3.09	2.58	11.25	16.15	1.98	1.74	0.29	0.15	4.33	4.28	1.25	1.35	2.26	2.36	58.6	52.3
diet/exercise	M	45	46	T2D	T2D	170	98	95	33.9	32.9	9.63	5.88	8	7.5	388	168	3.14	1.69	0.66	0.61	9.83	3.33	1.04	0.97	0.26	0.23	3.9	3.84	1.22	1.35	2.28	2.31	41.2	37.5
Mean		50.25	51.25			178	108.125	105.75	34.0	33.3	8.71625	6.69375	7.3375	7.1375	189.5125	141.013	1.83625	1.3625	1.33	1.33375	7.32875	5.015	1.69125	1.62875	0.44	0.37625	4.40375	4.43	1.29625	1.3675	2.505	2.5	55.5	53.1753
Student´s T-test p-value							0.0679		0.0636		0.0739		0.1666		0.1371		0.1244		0.9726		0.3309		0.1344		0.2996		0.5158		0.1867		0.9454		0.1459	

Intervention	Gender	Age		Diabetes		Height (cm)	Body weight (kg)		BMI (kg/m²)		Fasting plasma glucose (mmol/l)		HbA1c (%)		Fasting plasma insulin (pmol/l)		C-Peptid (nmol/l)		gGT (µkat/l)		CRP (mg/l)		Triglycerides (mmol/l)		FFA (mmol/l)		Cholesterol total (mmol/l)		HDL-Cholesterol (mmol/l)		LDL-Cholesterol (mmol/l)		sFNDC4 ng/ml	
	Baseline	Baseline	12 months	Baseline	12 months	Baseline	Baseline	12 months	Baseline	12 months	Baseline	12 months	Baseline	12 months	Baseline	12 months	Baseline	12 months	Baseline	12 months	Baseline	12 months	Baseline	12 months	Baseline	12 months	Baseline	12 months	Baseline	12 months	Baseline	12 months	Baseline	12 months
diet/exercise	F	52	53	T2D	T2D	173	119	105	39.8	35.1	5.45	6.94	6.30	6.50	144.1	103.8	1.98	1.61	0.69	0.78	0.81	1.44	1.4	1.45	0.69	0.38	5.21	5.22	0.98	1.09	3.39	3.11	52.8	52.9
diet/exercise	F	51	52	T2D	T2D	165	95	93	34.9	34.2	6.35	5.99	7.80	7.50	623.0	238.0	N/A	1.99	0.44	0.49	2.03	6.97	1.73	1.59	0.53	0.35	5.05	5.1	1.11	1.58	3.5	3.48	73.9	58.2
diet/exercise	F	54	55	T2D	T2D	164	91	92	33.8	34.2	6.99	6.05	6.40	6.10	493.6	30.0	0.19	1.19	0.35	0.33	0.84	5.03	1.39	1.28	0.54	0.51	4.14	3.95	1.31	1.32	2.38	2.4	51.4	50.0
diet/exercise	F	48	49	T2D	T2D	160	87	83	34.0	32.4	8.51	7.24	6.30	6.20	114.0	124.1	1.06	1.87	1.02	0.99	3.78	0.66	0.83	0.74	0.35	0.32	2.24	2.18	0.99	1.19	0.93	1.29	134.6	105.8
diet/exercise	F	53	54	T2D	T2D	159	82	80	32.4	31.6	7.16	13.44	6.90	5.90	55.5	146.8	0.81	1.29	0.72	0.72	2.77	1.67	0.77	0.92	0.84	0.51	4.49	4.28	1.8	1.66	2.27	2.22	59.3	42.3
diet/exercise	F	51	52	T2D	T2D	180	105	101	32.4	31.2	10.95	6.44	7.30	6.30	135.9	185.5	1.71	2.32	1.14	1.06	3.11	3.84	1.45	1.32	0.32	0.09	4.26	4.62	0.91	0.95	2.72	3.02	51.6	48.7
diet/exercise	F	49	50	T2D	T2D	164	99	95	36.8	35.3	13.79	6.92	8.80	8.50	180.6	108.0	2.15	1.83	0.61	0.53	3.82	5.28	1.22	1.15	0.79	0.8	5.66	5.81	1.6	1.32	3.41	2.99	51.9	54.6
diet/exercise	F	52	53	T2D	T2D	160	101	102	39.5	39.8	7.97	7.06	7.40	6.90	194.0	143.1	1.2	1.02	1.44	1.93	2.74	2.43	2.41	2.36	0.76	0.77	6.18	5.88	1.56	1.95	3.79	4.07	65.6	63.1
diet/exercise	F	54	55	T2D	T2D	162	85	86	32.4	32.8	9.52	6.69	8.00	7.80	30.0	19.6	0.35	0.41	2.45	1.93	7.87	3.19	2.3	2.9	0.9	0.85	4.49	4.52	0.96	0.84	2.93	2.48	57.4	59.2
diet/exercise	F	46	47	T2D	T2D	162	99	97	37.7	37.0	6.57	7.52	6.90	6.90	85.2	63.1	1.07	0.76	0.81	0.75	3.65	1.65	2.81	1.82	0.84	0.73	5.98	6.2	1.01	0.93	4.28	4.52	48.1	51.3
diet/exercise	F	49	50	T2D	T2D	165	119	118	43.7	43.3	4.65	8.50	6.80	6.50	73.2	176.6	0.98	1.95	2.29	2.38	8.51	0.41	1.42	1.27	0.12	0.11	4.51	4.44	1.52	1.66	2.72	2.89	64.7	64.5
diet/exercise	F	51	52	T2D	T2D	180	128	130	39.5	40.1	13.3	6.13	7.00	7.20	148.3	307.3	0.5	3.16	0.44	0.44	9.81	1.64	1.09	0.82	0.69	0.69	5.3	5.29	1.25	1.32	3.85	3.6	52.4	51.1
diet/exercise	F	51	52	T2D	T2D	164	110	109	40.9	40.5	6.79	7.12	6.10	6.20	354.3	267.9	2.87	2.46	0.47	0.44	1.73	1.18	2.07	1.82	0.38	0.41	6.08	6.1	1.37	1.39	3.84	3.84	57.4	50.3
diet/exercise	F	50	51	T2D	T2D	152	79	83	34.2	35.9	7.67	6.94	8.30	7.90	187.9	81.2	1.52	1.37	1.27	1.11	5.03	2.49	1.07	0.98	0.76	0.89	4.43	4.62	1.32	1.45	2.57	2.63	52.7	52.0
diet/exercise	F	48	49	T2D	T2D	178	106	99	33.5	31.2	7.84	6.95	6.60	6.30	121.5	93.9	1.18	0.887	0.54	0.46	8.56	4.92	0.89	0.75	0.81	0.62	5.29	5.3	1.57	1.49	3.2	3.1	49.4	50.1
Mean		50.6	51.6			165.87	100.33	98.20	36.36	35.65	8.23	7.33	7.13	6.85	196.07	139.26	1.26	1.61	0.98	0.96	4.34	2.85	1.52	1.41	0.62	0.54	4.89	4.90	1.28	1.34	3.05	3.04	61.54	56.95
Student´s T-test p-value							0.0767		0.0850		0.3407		0.0095		0.2093		0.1765		0.6707		0.1518		0.1957		0.0258		0.7691		0.2676		0.8899		0.0682	

Student´s T-test is a paired T-test

Supplementary Table 2 : Related to Figure 7a-g. Subjects information and metabolic parameters before and 12 months after the interventions :Bariatric surgery, Diet/exercise (Continued)

Intervention	Gender	Age		Diabetes		Height (cm)	Body weight (kg)		BMI (kg/m²)		Body fat (%)		Waist (cm)		Hip (cm)		WHR		Fasting plasma glucose (mmol/l)		HbA1c (%)		Fasting plasma insulin (pmol/l)		C-Peptid (nmol/l)		gGT (µkat/l)		CRP (mg/l)		Triglycerides (mmol/l)		FFA (mmol/l)		Cholesterol total (mmol/l)		HDL-Cholesterol (mmol/l)		LDL-Cholesterol (mmol/l)		sFNDC4 ng/ml	
	Baseline	Baseline	12 months	Baseline	12 months	Baseline	Baseline	12 months	Baseline	12 months	Baseline	12 months	Baseline	12 months	Baseline	12 months	Baseline	12 months	Baseline	12 months	Baseline	12 months	Baseline	12 months	Baseline	12 months	Baseline	12 months	Baseline	12 months	Baseline	12 months	Baseline	12 months	Baseline	12 months	Baseline	12 months	Baseline	12 months
bariatric surgery	M	42.0	43.0	no	no	188.0	200.0	144.6	56.6	40.9	34.8	34.3	186.0	139.0	159.0	121.0	1.2	1.1	5.9	5.6	6.1	5.3	495.4	143.2	N/A	1.6	1.2	0.6	2.6	0.7	1.5	1.3	0.7	0.7	4.1	5.4	1.1	1.1	2.4	3.7	52.1	58.1
bariatric surgery	M	45.0	46.0	no	no	188.0	183.0	138.1	51.8	39.5	40.6	36.4	162.0	140.0	165.0	136.0	1.0	1.0	5.7	5.5	6.3	5.3	285.1	144.1	2.4	2.0	0.7	0.4	8.9	4.8	1.5	1.5	0.4	0.5	5.8	5.7	1.5	1.7	3.8	3.4	51.7	57.4
bariatric surgery	M	53.0	54.0	no	no	183.0	174.0	112.7	52.0	33.7	44.0	32.5	132.0	116.0	140.0	126.0	0.9	0.9	5.6	4.7	6.4	4.9	350.4	73.2	2.7	1.0	1.7	1.7	6.3	2.8	3.9	1.4	0.9	0.2	6.2	5.4	0.9	1.2	3.4	3.7	46.9	49.7
bariatric surgery	M	44.0	45.0	no	no	189.0	214.0	137.0	59.9	38.4	45.1	29.2	168.0	129.0	143.0	122.0	N/A	N/A	5.6	5.0	5.6	5.2	65.3	42.1	0.7	1.0	0.4	0.3	4.3	2.6	1.3	1.1	0.3	0.2	4.2	4.2	1.5	1.3	2.2	2.4	44.5	41.9
bariatric surgery	M	28.0	29.0	no	no	185.0	209.0	121.0	60.9	35.2	52.1	N/A	178.0	144.0	169.0	125.0	N/A	N/A	5.9	5.3	5.7	5.2	63.2	78.5	0.7	0.8	0.9	0.5	4.7	2.1	0.9	0.8	0.2	0.2	3.8	3.8	1.1	1.2	2.2	2.3	40.9	41.2
Mean		42.4	43.4			186.6	196.0	130.7	56.2	37.5	43.3	33.1	165.2	133.6	155.2	126.0	1.0	1.0	5.8	5.2	6.0	5.2	251.9	96.2	1.6	1.3	1.0	0.7	5.4	2.6	1.8	1.2	0.5	0.3	4.8	4.9	1.2	1.3	2.8	3.1	47.2	49.7
Student´s T-test p-value							0.0011		0.0013		0.1053		0.0049		0.0059		0.9559		0.0134		0.0166		0.0930		0.4238		0.0876		0.0042		0.2753		0.4062		0.7548		0.3691		0.3968		0.2087	
Intervention	Gender	Age		Diabetes		Height (cm)	Body weight (kg)		BMI (kg/m²)		Body fat (%)		Waist (cm)		Hip (cm)		WHR		Fasting plasma glucose (mmol/l)		HbA1c (%)		Fasting plasma insulin (pmol/l)		C-Peptid (nmol/l)		gGT (µkat/l)		CRP (mg/l)		Triglycerides (mmol/l)		FFA (mmol/l)		Cholesterol total (mmol/l)		HDL-Cholesterol (mmol/l)		LDL-Cholesterol (mmol/l)		sFNDC4 ng/ml	
	Baseline	Baseline	12 months	Baseline	12 months	Baseline	Baseline	12 months	Baseline	12 months	Baseline	12 months	Baseline	12 months	Baseline	12 months	Baseline	12 months	Baseline	12 months	Baseline	12 months	Baseline	12 months	Baseline	12 months	Baseline	12 months	Baseline	12 months	Baseline	12 months	Baseline	12 months	Baseline	12 months	Baseline	12 months	Baseline	12 months
bariatric surgery	F	46.0	47.0	no	no	175.0	148.7	89.7	48.6	29.3	49.4	31.1	139.0	99.0	158.0	106.0	0.9	0.9	5.3	5.0	6.2	5.6	106.0	13.5	0.8	0.7	0.1	0.2	9.7	2.2	1.0	1.0	0.3	0.3	4.2	4.7	1.3	1.3	2.2	2.9	45.8	51.0
bariatric surgery	F	52.0	53.0	no	no	174.0	141.8	102.3	46.8	33.8	49.7	38.5	130.0	114.0	149.0	115.0	0.9	1.0	5.6	4.8	5.7	5.0	242.6	32.7	1.8	0.9	0.4	0.2	1.5	8.8	1.4	1.2	0.5	0.4	5.5	5.4	1.1	1.7	3.7	3.1	47.1	51.5
bariatric surgery	F	57.0	58.0	no	no	163.0	142.0	93.2	53.4	35.1	54.2	39.7	137.0	103.0	156.0	112.0	0.9	0.9	6.2	5.3	5.3	4.6	74.5	27.0	1.1	0.9	0.8	1.6	5.5	3.1	1.8	1.5	0.3	0.3	6.2	4.5	1.4	1.3	4.1	2.7	43.6	49.8
bariatric surgery	F	29.0	30.0	no	no	162.0	139.7	95.5	53.2	36.4	51.6	41.0	128.0	108.0	131.0	116.0	1.0	0.9	4.9	5.0	5.5	4.9	208.0	58.7	1.4	0.7	0.6	0.2	9.8	1.5	1.6	0.9	0.5	0.6	4.8	3.3	0.9	1.1	2.9	1.8	46.7	46.7
bariatric surgery	F	42.0	43.0	no	no	170.0	150.0	102.0	51.9	35.3	46.7	27.5	162.0	121.0	136.0	124.0	N/A	N/A	5.1	5.0	5.3	5.2	67.2	32.6	1.3	1.0	0.5	0.3	3.7	2.6	1.8	1.5	0.4	0.2	5.2	5.3	1.3	1.4	3.4	3.3	47.0	48.0
Mean		45.2	46.2			168.8	144.4	96.5	50.8	34.0	50.3	35.6	139.2	109.0	146.0	114.6	0.9	0.9	5.4	5.0	5.6	5.1	139.7	32.9	1.3	0.8	0.5	0.5	6.0	3.6	1.5	1.2	0.4	0.4	5.2	4.7	1.2	1.4	3.2	2.8	46.0	49.4
Student´s T-test p-value							0.0001		0.0001		0.0011		0.0043		0.0162		0.3027		0.0671		0.0069		0.0309		0.0420		0.9426		0.4389		0.0535		0.8814		0.2928		0.2225		0.2830		0.0505	
Intervention	Gender	Age		Diabetes		Height (cm)	Body weight (kg)		BMI (kg/m²)		Body fat (%)		Waist (cm)		Hip (cm)		WHR		Fasting plasma glucose (mmol/l)		HbA1c (%)		Fasting plasma insulin (pmol/l)		C-Peptid (nmol/l)		gGT (µkat/l)		CRP (mg/l)		Triglycerides (mmol/l)		FFA (mmol/l)		Cholesterol total (mmol/l)		HDL-Cholesterol (mmol/l)		LDL-Cholesterol (mmol/l)		sFNDC4 ng/ml	
	Baseline	Baseline	12 months	Baseline	12 months	Baseline	Baseline	12 months	Baseline	12 months	Baseline	12 months	Baseline	12 months	Baseline	12 months	Baseline	12 months	Baseline	12 months	Baseline	12 months	Baseline	12 months	Baseline	12 months	Baseline	12 months	Baseline	12 months	Baseline	12 months	Baseline	12 months	Baseline	12 months	Baseline	12 months	Baseline	12 months
bariatric surgery	M	50.0	51.0	T2D	no	182.0	135.0	80.0	40.7	24.1	42.8	32.5	139.0	114.0	140.0	113.0	N/A	N/A	10.3	5.0	6.9	5.9	95.0	44.3	1.0	0.7	1.0	0.9	6.7	3.5	1.4	1.2	0.3	0.2	6.0	5.9	1.3	1.3	4.0	4.2	56.5	65.2
bariatric surgery	M	54.0	55.0	T2D	no	179.0	156.0	110.0	48.7	34.3	35.8	26.1	143.0	116.0	150.0	113.0	N/A	N/A	6.4	5.2	8.3	5.8	161.0	25.4	1.6	0.6	0.9	0.6	1.7	4.9	0.6	0.6	0.6	0.2	3.9	4.0	1.4	1.5	1.9	2.1	69.2	76.0
bariatric surgery	M	44.0	45.0	T2D	no	190.0	169.0	115.0	46.8	32.2	33.6	N/A	152.0	121.0	149.0	124.0	1.0	1.0	8.0	4.3	6.5	4.5	461.5	22.7	2.7	1.6	0.7	0.4	9.7	4.1	4.0	1.6	0.9	0.2	4.6	4.6	0.7	1.0	2.7	3.0	52.3	63.4
bariatric surgery	M	51.0	52.0	T2D	no	178.0	152.0	109.7	48.0	34.6	37.7	30.7	145.0	121.0	142.0	129.0	1.0	0.9	5.8	5.1	7.3	4.6	245.6	75.1	2.9	0.6	3.1	1.0	5.2	1.5	2.6	1.4	0.3	0.6	5.8	4.4	1.1	1.4	4.0	2.5	60.9	69.5
bariatric surgery	M	57.0	58.0	T2D	no	174.0	180.3	107.6	59.6	35.5	41.3	26.7	160.0	124.0	166.0	130.0	1.0	1.0	8.0	5.0	6.7	5.0	251.0	52.3	N/A	0.5	0.9	0.5	4.6	3.2	2.1	0.8	0.9	0.4	3.6	4.7	1.1	2.2	1.8	2.2	46.6	56.7
bariatric surgery	M	54.0	55.0	T2D	no	190.0	195.0	121.0	54.0	33.5	N/A	N/A	175.0	152.0	180.0	148.0	1.0	1.0	6.8	5.3	5.9	4.6	130.1	5.3	1.5	0.9	1.5	1.0	9.8	1.3	1.1	1.1	0.1	0.2	5.0	5.8	1.9	2.3	3.0	2.9	50.6	58.0
bariatric surgery	M	44.0	45.0	T2D	no	171.0	129.0	86.7	44.1	29.7	38.5	28.8	140.0	125.0	122.0	131.0	1.1	1.0	5.8	5.1	6.3	4.6	124.8	22.7	1.8	0.7	3.4	1.0	16.3	7.1	3.5	1.0	0.2	0.3	7.3	3.9	1.3	1.1	4.7	2.2	53.6	59.3
bariatric surgery	M	50.0	51.0	T2D	no	186.0	150.0	98.0	43.4	28.3	40.8	27.9	137.0	110.0	146.0	116.0	N/A	N/A	12.6	5.0	9.5	5.8	48.6	50.6	0.7	0.3	0.1	0.1	0.8	5.8	0.7	0.7	0.4	0.4	3.0	2.6	1.4	1.6	1.4	1.5	58.4	39.8
bariatric surgery	M	48.0	49.0	T2D	no	186.0	175.9	118.6	50.8	34.3	36.8	25.6	163.0	126.0	170.0	130.0	1.0	1.0	10.9	6.1	8.6	5.8	134.5	4.1	2.3	0.4	0.5	0.3	7.9	1.6	4.8	1.8	0.8	0.5	4.6	4.1	0.7	1.0	2.3	2.4	53.2	55.0
Mean		50.2	51.2			181.8	160.2	105.2	48.5	31.8	38.4	28.3	150.4	123.2	151.7	126.0	1.0	1.0	8.3	5.1	7.3	5.2	183.6	33.6	1.8	0.7	1.3	0.6	7.0	3.7	2.3	1.1	0.5	0.3	4.9	4.4	1.2	1.5	2.9	2.6	55.7	60.3
Student´s T-test p-value							0.0000		0.0000		0.0000		0.0000		0.0010		0.2671		0.0040		0.0001		0.0067		0.0034		0.0457		0.0787		0.0189		0.2409		0.3518		0.0625		0.3758		0.1687	
Intervention	Gender	Age		Diabetes		Height (cm)	Body weight (kg)		BMI (kg/m²)		Body fat (%)		Waist (cm)		Hip (cm)		WHR		Fasting plasma glucose (mmol/l)		HbA1c (%)		Fasting plasma insulin (pmol/l)		C-Peptid (nmol/l)		gGT (µkat/l)		CRP (mg/l)		Triglycerides (mmol/l)		FFA (mmol/l)		Cholesterol total (mmol/l)		HDL-Cholesterol (mmol/l)		LDL-Cholesterol (mmol/l)		sFNDC4 ng/ml	
	Baseline	Baseline	12 months	Baseline	12 months	Baseline	Baseline	12 months	Baseline	12 months	Baseline	12 months	Baseline	12 months	Baseline	12 months	Baseline	12 months	Baseline	12 months	Baseline	12 months	Baseline	12 months	Baseline	12 months	Baseline	12 months	Baseline	12 months	Baseline	12 months	Baseline	12 months	Baseline	12 months	Baseline	12 months	Baseline	12 months
bariatric surgery	F	48.0	49.0	T2D	no	168.0	116.0	86.0	41.1	30.4	45.2	32.5	129.0	105.0	137.0	117.0	N/A	N/A	7.6	5.7	9.7	5.8	165.0	26.4	1.6	0.9	0.8	0.5	6.5	0.6	1.1	1.1	0.7	0.2	4.6	4.7	1.7	1.8	2.7	2.6	48.6	56.1
bariatric surgery	F	52.0	53.0	T2D	no	166.0	125.0	95.0	45.3	34.4	51.0	29.8	136.0	103.0	139.0	106.0	N/A	N/A	8.6	4.9	6.9	5.5	526.0	45.1	1.6	0.9	1.0	1.0	7.8	1.1	1.5	1.4	0.6	0.2	4.5	4.6	1.1	1.4	2.9	3.1	48.0	56.2
bariatric surgery	F	51.0	52.0	T2D	no	170.0	134.0	87.0	46.4	30.1	46.3	26.7	125.0	99.0	142.0	126.0	N/A	N/A	9.4	5.3	7.6	5.6	124.1	39.7	1.3	0.8	0.9	0.8	1.7	4.9	1.9	1.4	0.5	0.4	6.4	5.9	1.7	1.8	4.1	4.1	49.6	61.9
bariatric surgery	F	53.0	54.0	T2D	no	166.0	119.0	76.0	43.1	27.5	39.6	26.6	122.0	98.0	131.0	109.0	N/A	N/A	11.7	5.8	7.5	5.7	91.6	19.3	1.5	0.6	0.8	0.8	0.3	3.3	1.5	1.1	0.3	0.1	5.7	5.7	1.5	1.5	3.4	3.3	48.0	54.5
bariatric surgery	F	49.0	50.0	T2D	no	162.0	155.0	98.0	59.1	37.3	36.8	28.6	162.0	125.0	160.0	132.0	N/A	N/A	8.6	5.2	8.5	5.9	143.0	32.3	1.4	0.8	0.5	0.6	2.3	6.9	0.6	0.6	0.4	0.2	3.8	3.9	1.6	1.7	2.1	2.0	55.5	64.2
bariatric surgery	F	58.0	59.0	T2D	no	159.0	118.0	83.0	46.7	32.8	30.9	30.8	143.0	115.0	139.0	121.0	N/A	N/A	5.9	5.1	7.6	5.7	96.0	21.7	0.9	0.6	1.1	0.6	1.0	3.9	1.4	1.0	0.8	0.1	4.0	4.3	1.6	2.1	2.2	1.9	49.2	55.3
bariatric surgery	F	46.0	47.0	T2D	no	164.0	129.0	101.0	47.9	37.6	42.1	31.5	138.0	120.0	140.0	128.0	N/A	N/A	9.5	4.7	8.6	5.6	138.0	23.5	2.1	0.4	0.3	0.3	8.5	0.5	1.3	1.1	0.4	0.3	3.7	3.5	1.0	1.1	2.2	2.1	56.7	82.2
bariatric surgery	F	48.0	49.0	T2D	no	165.0	136.0	82.0	50.0	30.1	41.6	28.6	155.0	118.0	156.0	124.0	N/A	N/A	7.3	5.9	9.2	5.3	154.2	16.8	1.7	0.9	1.4	1.2	0.8	9.9	1.3	1.1	0.4	0.2	2.8	3.1	1.0	1.3	1.5	1.7	48.1	56.1
bariatric surgery	F	42.0	43.0	T2D	no	170.0	142.0	100.7	49.1	34.8	47.7	40.4	152.0	129.0	166.0	128.0	0.9	1.0	6.6	5.0	6.9	5.4	172.4	27.3	1.7	0.7	0.4	0.2	4.3	0.7	3.4	1.4	0.3	0.4	4.8	4.3	1.0	1.3	2.6	2.6	48.8	50.4
bariatric surgery	F	47.0	48.0	T2D	no	180.0	180.0	129.1	55.6	39.8	54.1	43.7	172.0	142.0	185.0	140.0	0.9	1.0	9.8	4.4	6.3	4.9	54.0	21.7	0.7	0.8	0.2	0.1	5.0	1.7	1.1	0.8	0.1	0.4	6.0	5.2	1.1	1.3	3.9	3.1	44.8	46.0
bariatric surgery	F	48.0	49.0	T2D	no	168.0	170.0	98.6	60.2	34.9	51.9	29.7	150.0	131.0	159.0	135.0	0.9	1.0	7.4	4.5	6.4	5.0	177.1	31.2	1.4	0.6	0.4	0.2	9.4	0.4	1.4	1.0	0.6	0.2	4.2	4.4	1.4	1.7	2.2	2.2	51.4	51.7
bariatric surgery	F	57.0	58.0	T2D	no	163.0	142.0	93.2	53.4	35.1	54.2	39.7	137.0	118.0	156.0	131.0	N/A	N/A	7.0	5.3	6.7	5.8	159.0	23.6	1.6	0.7	0.3	0.3	4.0	1.6	1.8	1.5	0.6	0.3	6.2	6.0	1.4	1.9	4.1	4.3	46.3	49.1
bariatric surgery	F	51.0	52.0	T2D	no	164.0	169.0	101.1	62.8	37.6	49.3	33.4	152.0	132.0	142.0	136.0	1.1	1.0	5.9	5.4	6.5	4.8	89.7	69.3	1.2	0.9	0.6	0.4	5.5	1.0	1.4	1.2	0.2	0.6	3.7	4.6	1.1	1.4	2.0	2.7	56.0	61.7
bariatric surgery	F	39.0	40.0	T2D	no	182.0	159.1	103.7	48.0	31.3	45.8	32.2	145.0	115.0	152.0	121.0	1.0	1.0	8.6	4.9	8.5	5.2	206.6	33.4	1.7	0.7	1.8	0.6	2.7	0.7	1.5	1.0	0.7	0.5	4.3	5.1	1.0	1.4	2.7	3.0	50.5	53.2
bariatric surgery	F	53.0	54.0	T2D	no	161.0	101.8	70.9	39.3	27.4	45.7	26.2	115.0	106.0	135.0	106.0	0.9	1.0	6.9	5.2	5.7	4.6	90.2	16.0	1.2	0.9	1.9	0.4	1.6	0.3	1.8	1.0	0.4	0.3	5.9	4.2	1.9	2.4	3.2	1.6	44.0	49.9
bariatric surgery	F	62.0	63.0	T2D	no	156.0	140.0	108.4	57.5	44.5	49.5	44.0	138.0	112.0	145.0	120.0	1.0	0.9	10.9	11.0	8.1	5.6	370.9	235.5	0.6	1.3	0.3	0.2	1.4	0.7	3.6	1.8	0.6	0.8	9.6	3.7	1.6	1.6	6.7	1.6	53.8	61.0
bariatric surgery	F	39.0	40.0	T2D	no	155.0	113.0	75.4	47.0	31.4		30.5	119.0	104.0	129.0	120.0	0.9	0.9	6.7	4.8	6.5	5.5	135.3	43.0	1.4	1.3	0.6	0.2	6.1	0.4	1.8	0.7	0.6	0.7	5.8	3.6	1.3	1.5	3.8	1.8	57.2	69.1
Mean		49.6	50.6			165.8	138.2	93.5	50.2	33.9	45.7	32.6	140.6	116.0	147.8	123.5	0.9	1.0	8.1	5.5	7.5	5.4	170.2	42.7	1.4	0.8	0.8	0.5	4.0	2.3	1.7	1.1	0.5	0.3	5.0	4.5	1.3	1.6	3.1	2.6	50.4	57.6
Student´s T-test p-value							0.000		0.000		0.000		0.000		0.000		0.481		0.000		0.000		0.000		0.000		0.015		0.156		0.002		0.036		0.187		0.000		0.156		0.000	
Intervention	Gender	Age		Diabetes		Height (cm)	Body weight (kg)		BMI (kg/m²)		Body fat (%)		Waist (cm)		Hip (cm)		WHR		Fasting plasma glucose (mmol/l)		HbA1c (%)		Fasting plasma insulin (pmol/l)		C-Peptid (nmol/l)		gGT (µkat/l)		CRP (mg/l)		Triglycerides (mmol/l)		FFA (mmol/l)		Cholesterol total (mmol/l)		HDL-Cholesterol (mmol/l)		LDL-Cholesterol (mmol/l)		sFNDC4 ng/ml	
	Baseline	Baseline	12 months	Baseline	12 months	Baseline	Baseline	12 months	Baseline	12 months	Baseline	12 months	Baseline	12 months	Baseline	12 months	Baseline	12 months	Baseline	12 months	Baseline	12 months	Baseline	12 months	Baseline	12 months	Baseline	12 months	Baseline	12 months	Baseline	12 months	Baseline	12 months	Baseline	12 months	Baseline	12 months	Baseline	12 months
bariatric surgery	M	65.0	66.0	T2D	T2D	180.0	132.0	95.3	40.7	29.3	45.5	24.2	N/A	N/A	N/A	N/A	N/A	N/A	13.4	7.2	7.1	7.1	76.9	105.3	1.4	1.6	1.4	0.9	5.2	3.5	1.5	1.3	0.7	0.4	5.7	4.7	1.5	2.4	3.4	2.2	56.3	47.8
bariatric surgery	M	46.0	47.0	T2D	T2D	178.0	121.4	108.0	38.3	34.1	30.7	28.5	125.0	118.0	110.0	106.0	N/A	N/A	9.0	9.5	7.6	7.5	365.0	211.0	1.6	1.5	1.2	1.1	6.8	5.7	2.0	1.8	1.3	0.8	3.8	4.0	1.1	1.0	2.5	2.7	49.6	50.4
bariatric surgery	M	52.0	53.0	T2D	T2D	185.0	156.0	132.0	45.5	38.5	42.3	35.6	141.0	103.0	132.0	98.0	N/A	N/A	9.3	8.5	9.7	7.9	326.0	158.0	N/A	N/A	0.8	0.8	7.2	5.1	2.1	2.0	0.7	0.8	4.0	4.2	1.0	1.0	2.5	2.2	37.1	35.2
bariatric surgery	M	44.0	45.0	T2D	T2D	185.0	144.0	108.0	42.0	31.6	33.5	23.1	N/A	N/A	N/A	N/A	N/A	N/A	8.7	12.8	8.0	8.2	563.0	429.0	N/A	N/A	0.9	0.9	9.7	8.3	2.5	2.3	1.0	0.9	4.8	5.0	0.9	0.9	2.9	3.2	73.6	53.1
bariatric surgery	M	48.0	49.0	T2D	T2D	180.0	185.0	136.0	57.1	42.0	40.1	29.4	158.0	122.0	149.0	118.0	N/A	N/A	8.0	8.6	7.9	6.9	101.5	86.5	1.5	0.3	0.8	0.5	2.1	0.9	1.2	1.1	0.2	0.2	5.1	4.8	1.5	1.6	3.4	3.3	53.6	55.8
bariatric surgery	M	50.0	51.0	T2D	T2D	175.0	163.0	105.0	53.2	34.2	39.8	39.6	163.0	127.0	169.0	131.0	N/A	N/A	9.4	6.7	8.6	7.9	55.2	56.2	0.8	1.3	0.6	0.3	2.5	3.9	1.3	1.1	0.8	0.9	4.5	4.9	1.6	1.7	2.6	2.4	56.7	52.0
Mean		50.8	51.8			180.5	150.2	114.1	46.1	35.0	38.7	30.1	146.8	117.5	140.0	113.3			9.6	8.9	8.1	7.6	247.9	174.3	1.3	1.2	1.0	0.7	5.6	4.6	1.8	1.6	0.8	0.7	4.7	4.6	1.2	1.4	2.9	2.7	54.5	49.0
Student´s T-test p-value							0.003		0.004		0.038		0.029		0.040				0.632		0.135		0.095		0.722		0.027		0.104		0.000		0.288		0.769		0.316		0.403		0.170	
Intervention	Gender	Age		Diabetes		Height (cm)	Body weight (kg)		BMI (kg/m²)		Body fat (%)		Waist (cm)		Hip (cm)		WHR		Fasting plasma glucose (mmol/l)		HbA1c (%)		Fasting plasma insulin (pmol/l)		C-Peptid (nmol/l)		gGT (µkat/l)		CRP (mg/l)		Triglycerides (mmol/l)		FFA (mmol/l)		Cholesterol total (mmol/l)		HDL-Cholesterol (mmol/l)		LDL-Cholesterol (mmol/l)		sFNDC4 ng/ml	
	Baseline	Baseline	12 months	Baseline	12 months	Baseline	Baseline	12 months	Baseline	12 months	Baseline	12 months	Baseline	12 months	Baseline	12 months	Baseline	12 months	Baseline	12 months	Baseline	12 months	Baseline	12 months	Baseline	12 months	Baseline	12 months	Baseline	12 months	Baseline	12 months	Baseline	12 months	Baseline	12 months	Baseline	12 months	Baseline	12 months
bariatric surgery	F	35.0	36.0	T2D	T2D	150.0	162.0	113.0	72.0	50.2	52.9	48.7	164.0	138.0	182.0	141.0	0.9	1.0	6.1	9.2	6.4	8.1	263.8	61.9	0.9	0.9	0.4	0.4	4.6	2.8	1.7	3.4	0.5	0.3	5.4	7.3	1.1	1.2	3.4	5.1	50.4	53.1
bariatric surgery	F	61.0	62.0	T2D	T2D	168.0	135.0	99.8	47.8	35.4		48.2	148.0	109.0	161.0	111.0	0.9	1.0	8.1	7.3	7.7	6.1	278.2	85.1	0.6	1.0	0.9	0.3	9.7	1.4	2.8	1.6	0.7	0.3	6.2	6.3	1.2	1.4	3.8	4.2	50.6	47.1
bariatric surgery	F	37.0	38.0	T2D	T2D	160.0	153.2	91.0	59.8	35.5	52.8	36.3	155.0	125.0	132.0	119.0	N/A	N/A	7.3	7.0	7.5	7.3	165.0	184.0	1.6	155.0	0.6	0.4	5.0	5.0	2.1	2.0	0.8	0.6	4.4	4.6	1.8	2.1	2.3	2.5	48.1	51.6
bariatric surgery	F	48.0	49.0	T2D	T2D	160.0	71.1	72.0	27.8	28.1	29.6	30.2	126.0	129.0	105.0	104.0	N/A	N/A	8.4	7.6	9.0	8.3	265.0	136.0	2.1	1.8	0.4	0.4	5.1	2.6	2.0	1.6	0.7	0.5	5.3	5.7	2.0	2.1	2.7	2.7	50.1	46.9
bariatric surgery	F	28.0	29.0	T2D	T2D	182.0	233.0	162.0	70.3	49.3	57.7	48.1	164.0	120.0	190.0	142.0	N/A	N/A	7.0	6.6	6.4	6.5	79.5	98.6	1.2	1.1	0.5	0.6	5.7	4.1	1.8	1.8	0.9	0.7	4.8	4.9	1.1	1.0	2.7	2.6	53.0	55.9
bariatric surgery	F	46.0	47.0	T2D	T2D	155.0	132.0	95.0	54.9	39.5	32.8	32.6	155.0	124.0	151.0	109.0	N/A	N/A	6.3	6.3	7.2	7.1	73.0	105.9	1.6	0.4	0.2	0.1	4.6	0.3	0.8	0.8	0.1	0.2	4.3	4.3	2.0	2.2	2.1	2.2	47.7	47.6
bariatric surgery	F	49.0	50.0	T2D	T2D	164.0	171.0	122.0	63.6	45.4	42.8	35.6	162.0	116.0	143.0	112.0	N/A	N/A	11.4	6.1	6.9	7.2	95.6	32.6	N/A	1.1	0.5	0.3	0.7	0.9	1.5	1.4	0.3	0.1	5.1	4.6	1.4	2.5	3.3	3.3	66.1	61.2
bariatric surgery	F	51.0	52.0	T2D	T2D	162.0	128.0	78.0	48.8	29.7	36.7	25.6	134.0	112.0	125.0	109.0	N/A	N/A	13.8	7.2	8.2	8.1	56.3	84.5	1.7	0.6	0.6	0.5	0.8	5.5	2.5	2.0	0.2	0.1	5.5	5.3	0.9	1.0	3.7	3.8	55.9	53.1
Mean		44.4	45.4			162.6	148.2	104.1	55.6	39.1	43.6	38.2	151.0	121.6	148.6	118.4	0.9	1.0	8.5	7.2	7.4	7.3	159.6	98.6	1.4	20.2	0.5	0.4	4.5	2.8	1.9	1.8	0.5	0.3	5.1	5.4	1.4	1.7	3.0	3.3	52.7	52.0
Student´s T-test p-value							0.001		0.001		0.025		0.001		0.002		0.067		0.253		0.812		0.132		0.363		0.120		0.243		0.780		0.002		0.381		0.107		0.191		0.573	

Student´s T-test is a paired T-test

Supplementary Table 3: List of all used primer sequences
m:mouse, h: human

Gene name	TaqMan probe ID	Species
hFNDC4	Hs01100278_g1	human
hGPR116	Hs00391810_m1	human
18S rRNA	Hs99999901_s1	human

Gene name	5´to 3´primer sequence	Species
mGpr116-F	GAT CGC GTG GCG GAA GAA TA	mouse
mGpr116-R	GTC CCT CGA ATT GGA AAA	mouse
hGPR116-F	AGACAGGTTATGGGTGGCCT	human
hGPR116-R	TCCAAGTCGGTCTTGTAGGAC	human
hRXFP1-F	TATGGCACCAATGGAGTATGCT	human
hRXFP1-R	ACTATGATGATAAATGCGGCCAA	human
mTbp-F	GAGCCAAGAGTGAAGAACAGTC	mouse
mTbp-R	GCTCCCCACCATATTCTGAATCT	mouse
hTBP-F	CCACTCACAGACTCTCACAAC	human
hTBP-R	CTGCGGTACAATCCCAGAACT	human
mFndc4-F	ACCTCCCTCTCCTGTGAATGT	mouse
mFndc4-R	ATGGAGTAGCCAATGACGATG	mouse
mHprt -F	AGTCCCAGCGTCGTGATTAG	mouse
mHprt-R	TTTCCAAATCCTCGGCATAATGA	mouse
mBeta actin - F	GTGACGTTGACATCCGTAAAGA	mouse
mBeta actin -R	GCCGGACTCATCGTACTCC	mouse
mIL6-F	CACGCAGACTTCATCCTCCACA	mouse
mIL6-R	AGCTATAGTCCATCCGTGCCTG	mouse
mIL1b-F	CAAGCAATACCCAAAGAAGA	mouse
mIL1b-R	GAAACAGTCCAGCCCATAC	mouse
mMcp1-F	GCCTGCTGTTCACAGTTG	mouse
mMcp1-R	CATCTTGCTGGTGAATGAGTA	mouse
mCd206-F	AAATGGTCAGAGGCACAGTTC	mouse
mCd206-R	GGAGGCTGGCTGTGATAAATG	mouse
mIL10-F	GGAGCAGGTGAAGAGTGATT	mouse
mIL10-R	AGCTCTGTCTAGGTCCTGGA	mouse
mCcl24-F	AATTCCAGAAAACCGAGTG	mouse
mCcl24-R	TCCAGTTTTTGTATGTGCC	mouse
mCcl11-F	GAAGATCCCCAACACACTACTG	mouse
mCcl11-R	GATCTCTTTGCCCAACCTG	mouse
mCd68-F	TGTCTGATCTTGCTAGGACCG	mouse
mCd68-R	GAGAGTAACGGCCTTTTTGTGA	mouse
mTNFalpha-F	CCACCACGCTCTTCTGTCTAC	mouse
mTNFalpha-R	GGTCTGGGCCATAGAACTGAT	mouse
mResistin-F	AAGAACCTTTCATTTCCCCTCCT	mouse
mResistin-R	GTCCAGCAATTTAAGCCAATGTT	mouse
mH3f3-F	TGTGGCCCTCCGTGAAATC	mouse
mH3f3-R	GGCATAATTGTTACACGTTTGGC	mouse
mEdem-F	AGTCAAATGTGGATATGCTACGC	mouse
mEdem-R	ACAGATATGATATGGCCCTCAGT	mouse
mXbp-1-F	GACAGAGAGTCAAACTAACGTGG	mouse
mXbp1-R	GTCCAGCAGGCAAGAAGGT	mouse
mAtf6-F	GTCCAAAGCGAAGAGCTGTCTG	mouse
mAtf6-R	AGAGATGCCTCCTCTGATTGGC	mouse
mHmox-1-F	AGGGTCAGGTGTCCAGAGAA	mouse
mHmox-1-R	CTTCCAGGGCCGTGTAGATA	mouse
mDNAjb9-F	ATAAAAGCCCTGATGCTGAAGC	mouse
mDNAjb9-R	GCCATTGGTAAAAGCACTGTGT	mouse
mGrp78-F	TGTCTTCTCAGCATCAAGCAAGG	mouse
mGrp78-R	CCAACACTTCCTGGACAGGCTT	mouse
mGrp94-F	CTCACAGAGCCTGTGGATGA	mouse
mGrp94-R	TCTCTGTTGCTTCCCGACTT	mouse
mERdj4-F	CTCCACAGTCAGTTTTCGTCTT	mouse
mERdj4-R	GGCCTTTTTGATTTGTCGCTC	mouse
mChop-F	CTGGAAGCCTGGTATGAGGAT	mouse
mChop-R	CAGGGTCAAGAGTAGTGAAGGT	mouse

Genotyping primers	5´to 3´primer sequence	Species
GPR116-F	GGAGGCTCTGTGCGTTTC	mouse
GPR116-R1	CTGTGGACATGATGAAGGGTG	mouse
GPR116-R2	CTCCCTGAATCATAGTCTAGTCTCC	mouse
Cre-1	GAACCTGATGGACATGTTCAGG	mouse
Cre-2	AGTGCGTTCGAACGCTAGAGCCTGT	mouse
Cre-3	TTACGTCCATCGTGGACAGC	mouse
Cre-4	TGGGCTGGGTGTTAGCCTTA	mouse
