# Supplementary material for: Orphan GPR116 mediates the insulin sensitizing effects of the hepatokine FNDC4 in adipose tissue
Source: Nat Commun. 2021 May 20;12:2999. doi: 10.1038/s41467-021-22579-1 (PMC8137956; doi:10.1038/s41467-021-22579-1)
Supplement: Supplementary file 3 — Reporting Summary [file 41467_2021_22579_MOESM3_ESM.pdf]

nature research

Corresponding author(s): Prof.Dr.Stephan Herzig, Dr. Anastasia Georgiadi

Reporting Summary

Nature Research wishes to improve the reproducibility of the work that we publish. This form provides structure for consistency and transparency in reporting. For further information on Nature Research policies, see Authors & Referees and the Editorial Policy Checklist.

Statistical parameters

When statistical analyses are reported, confirm that the following items are present in the relevant location (e.g. figure legend, table legend, main text, or Methods section).

n/a	Confirmed	
☐	☒	The exact sample size (n) for each experimental group/condition, given as a discrete number and unit of measurement
☐	☒	An indication of whether measurements were taken from distinct samples or whether the same sample was measured repeatedly
☐	☒	The statistical test(s) used AND whether they are one- or two-sided<br>*Only common tests should be described solely by name; describe more complex techniques in the Methods section.*
☐	☒	A description of all covariates tested
☐	☒	A description of any assumptions or corrections, such as tests of normality and adjustment for multiple comparisons
☐	☒	A full description of the statistics including central tendency (e.g. means) or other basic estimates (e.g. regression coefficient) AND variation (e.g. standard deviation) or associated estimates of uncertainty (e.g. confidence intervals)
☐	☒	For null hypothesis testing, the test statistic (e.g. F, t, r) with confidence intervals, effect sizes, degrees of freedom and P value noted<br>*Give P values as exact values whenever suitable.*
☒	☐	For Bayesian analysis, information on the choice of priors and Markov chain Monte Carlo settings
☒	☐	For hierarchical and complex designs, identification of the appropriate level for tests and full reporting of outcomes
☐	☒	Estimates of effect sizes (e.g. Cohen's d, Pearson's r), indicating how they were calculated
☐	☒	Clearly defined error bars<br>*State explicitly what error bars represent (e.g. SD, SE, CI)*

Our web collection on statistics for biologists may be useful.

Software and code

Policy information about availability of computer code

Data collection	Image Lab (Bio-rad) version 5.2.1 build 11 was use to collect Western blot images
Data analysis	GraphPad prism v.7 was used for graphs generation and statistical analysis

For manuscripts utilizing custom algorithms or software that are central to the research but not yet described in published literature, software must be made available to editors/reviewers upon request. We strongly encourage code deposition in a community repository (e.g. GitHub). See the Nature Research guidelines for submitting code & software for further information.

Data

Policy information about availability of data

All manuscripts must include a data availability statement. This statement should provide the following information, where applicable:

- Accession codes, unique identifiers, or web links for publicly available datasets
- A list of figures that have associated raw data
- A description of any restrictions on data availability

Source data are provided with this paper. Mouse models are available with MTA agreement. All data supporting the findings are available within the article and its supplementary information files and from the corresponding authors upon request.
Microarray data are available in GEOprofiles under the number: GSE165329 (https://www.ncbi.nlm.nih.gov/geo/query/acc.cgi?acc=GSE165329). pTPM data for

FNDC4 expression are available from Protein Atlas and exact URLs for each tissue are included in the SourceData.

Field-specific reporting

Please select the best fit for your research. If you are not sure, read the appropriate sections before making your selection.

☒ Life sciences ☐ Behavioural & social sciences ☐ Ecological, evolutionary & environmental sciences

For a reference copy of the document with all sections, see nature.com/authors/policies/ReportingSummary-flat.pdf

Life sciences study design

All studies must disclose on these points even when the disclosure is negative.

Sample size	No exact calculations or formula was used for sample calculations. For in vivo experiments at least n= 5-8 was chosen in order to detect moderate to large (>20%) differences, a reproducible way. This was estimated by magnitude of effects during pilot experiments. For in vitro experiment as a default at least 3 replicate wells were used per group in the same experiment. Significance of differences in data was determined by two- tailed Student´s t-test, unpaired or paired or by ANOVA , multiple comparisons as indicated in the figure legends.
Data exclusions	Cell studies; No data exclusion, Animal studies: No data exclusion upon completion of the protocol.
Replication	We utilized in vitro several replicates well (technical replicates) and biological replicates and performed experiments more than once and our findings were reproducible. We have used different assays, in vitro and in vivo model in order to verify to assess our hypotheses.
Randomization	For animal studies mice covariate such as age, sex and body weight were at first used to select mice of same age, sex and body weight (at the start of the experiment) and those mice were randomly allocated into treatment groups before the initiation of the study. For human intervention study (NUGAT) the glucose tolerance at baseline was used as a covariant of including individuals as part of the study. All individuals that have fulfilled this criteria at baseline were then randomly assigned to diet groups. For the Weight loss intervention study (Leipzig) all individuals that entered the study were obese according to BMI definitions >28kg/m2 and underwent weight loos intervention either bariatric surgery or diet /exercise. All individuals were categorized based on the existence or not of Type 2 diabetes at the time of the surgery and they were allocated into groups of T2D and no T2D. The categorization was base on three criteria: a) the requirement of anti-diabetic complications, b) HbA1c < 6%, c) fasting plasma glucose < 6mmol/l.
Blinding	Researchers were just partially blinded during the cell or animal studies performance, data collection and data analysis. Blinding was not possible because the same researcher who performed treatments on animals or cells was the same person collecting the data or at least was involved in data collection. Usually the person that ´runs´ the project performs the experiments and collects the data, in some cases with assistance from other researchers, so inevitably this person knows the identity of the study groups. For collection of histology images blinding was performed, as the person that collected the images was different from the person who took care of the collection of the tissues and their histological preparation.

Reporting for specific materials, systems and methods

Materials & experimental systems

n/a	Involved in the study
☐	☒ Unique biological materials
☐	☒ Antibodies
☐	☒ Eukaryotic cell lines
☒	☐ Palaeontology
☐	☒ Animals and other organisms
☐	☒ Human research participants

Methods

n/a	Involved in the study
☒	☐ ChIP-seq
☐	☒ Flow cytometry
☒	☐ MRI-based neuroimaging

Unique biological materials

Policy information about availability of materials

Obtaining unique materials	All unique materials are available upon request to the corresponding authors

Antibodies

Antibodies used

Anti-GPCR GPR116 antibody (ab136262)Host species: rabbit, reacts with : human, WB, working dilution 1:1000

anti-GPR116, Abcam cat. number ab111169, lot.GR48909-3, Host species: rabbit, reacts with : human, application: WB, working dilution 1:1000. For in vitro receptor blocking on live cells we used it at a concentration of 0,4ug/uL.

anti-beta tubulin, Sigma cat,number T5201, Host species: mouse, reactivity:
wheat, mouse, hamster, sea urchin, frog, plant, bovine, rat, rabbit, moth, chicken, human , application: WB, working dilution 1:1000

Recombinant Anti-VCP antibody [EPR3307(2)] (ab109240), Host: rabbit, Reactivity: mouse, rat, human, Application: WB, IP, IHC-P, Flow Cyt, ICC/IF, working dilution 1:1000
anti-beta actin Sigma Adlrich Cat.# A5441, host.: mouse, species reactivity: pig, Hirudo medicinalis, bovine, rat, canine, feline, human, rabbit, carp, mouse, guinea pig, chicken, sheep, application: WB, working dilution 1:1000
anti-pAS160(Thr642) Cell Signaling Technology Cat.# 4288, Host : rabbit, Species reactivity H, M , application: WB , working dilution 1:1000
anti-AS160 Cell Signaling Technology Cat.# 2670S, host: rabbit . Species reactivity : Human, mouse, rat, application: WB, working dilution 1:1000
anti-AKT Cell Signaling Technology Cat.# 9272, Source: Rabbit, Reactivity: Human, mouse, rat , Hm Mk C Dm B Dg Pg GP, Application : WB, working dilution 1:1000
anti-phopsho-PKA substrate Cell Signaling Technology Cat.# 9624, Host: Rabbit, Reactivity: All, Application: WB, working dilution 1:1000
anti-pCREB(Ser133) Cell Signaling Technology Cat.# 9198, Host: Rabbit, Reactivity: Human, Mouse, Rat, Application: WB, working dilution 1:1000
anti-pERK1/2 (T202/Y204) Cell Signaling Technology , cat.no 9101 lot.28, Host: Rabbit, Reactivity: Human, mouse ,rat, Hm Mk Mi Dm Z B Pg Ce, Application : WB, working dilution 1:1000
anti-pAMPKa(Thr172) Cell Signaling Technology Cat.#2535, Source: Rabbit, Reactivity: Human, mouse , rat , Hm Mk Dm Sc, Application: WB , working dilution 1:1000
Phospho-Stat3 (Tyr705) (D3A7) XP® Rabbit mAb #9145 , Host: Rabbit, Reactivity: Human, mouse, rat, monkey, Application: WB, working dilution 1:1000
anti-HO-1 Cell Signaling Technology Cat.# 43966, Host: Rabbit, Reactivity: Human, Mouse, Rat, Application : WB, working dilution 1:1000
pStat3 (phospho Y705) cell signalling 9145. Host: Rabbit, Reactivity: Human, mouse, rat , monkey, Application: WB, working dilution 1:1000

anti-STAT3 Cell Signaling Technology Cat.#12640. Host: rabbit, Reactivity: Human, mouse, rat, monkey, Application : WB, working dilution 1:1000
anti-phopsho-PKC substrate Cell Signaling Technology Cat.#6967, Host: rabbit, Reactivity: All, Application : WB, working dilution 1:1000
anti-ERK1/2, Cell signaling ,cat, no 4695, lot. no 21, Host: rabbit, Reactivity: All, Applicaton: WB, working dilution 1:1000
anti-F(ab')2-Goat anti-Human IgG Fc Secondary Antibody, PE, Invitrogen, cat. no H10104, lot no. 1814018A, 1683265C, Host : Goat, Reactivity: Human, Application: Flow cytometry, working Dilution : 1:200
polyclonal rabbit anti-CD68 antibody, #125212, Abcam, host: rabbit, reactivity: Mouse, rat, Applications: WB, IHC-P, , IHC-Fr., working dilution: 1:500
Secondary antibody: goat anti rabbit-biotinylated, Vector BA-1000, dilution 1:750
DAB Detection of the signal followed by the DAB Map Kit from Ventana Medical Systems
Rabbit IgG, polyclonal - Isotype Control (ChIP Grade) (ab171870) abcam Cat.# 171870, Applications: WB, ChIP, Flow cytometry. , working concentration: 0,4ug/uL for in vitro assays.
anti-rabbit, HRP conjugated , DAKO, P0399, Host: Pig, Reactivity: Rabbit, Applications: WB, Elisa, Radioimmunoassay, Immunihistochemistry, working dilution ; 1:10000.
anti-mouse, HRP conjugated, DAKO, P0447, Host: Goat, Reactivity: mouse, Application: WB, Elisa, Immuhistochemistry, working dilution ; 1:10000
secondary antibody: goat anti-rabbit IgG antibody (H+L), biotinylated, BA-1000, Vector, Germany, working dilution ; 1:1000
Host: goat, Reactivity: rabbit, Applications: Blocking Reagent, Primary Antibody (Rabbit IgG) or Negative Control Reagent, Biotinylated Secondary Antibody (Gt X Rb IgG),Streptavidin-Enzyme Conjugate, Chromogen Counterstain. working dilution ; 1:1000
anti-FNDC4 Sigma Adlrich Cat. # SAB1401807, host : rabbit, reactivity : human, working dilution 1:1000
Monoclonal ANTI-FLAG® M2-Peroxidase (HRP) Sigma Adlrich Cat. # A8592, source : mouse , reactivity : all, working dilution : 1:1000
ELISA: Anti-FNDC4 ELISA EK-067-90 from Phoenix Pharmaceuticals

anti-pAKT(Ser473) Cell Signaling Technology Cat.# 9271S, source: rabbit, reactivity : M R Hm Dm B Dg Pg, wB, working dilution 1:1000

Validation

-Anti-GPCR GPR116 antibody (ab136262) Validation: Wang C et al. MiR-511-5p functions as a tumor suppressor and a predictive of prognosis in colorectal cancer by directly targeting GPR116. Eur Rev Med Pharmacol Sci 23:6119-6130 (2019) and also in this paper we have validation data on mouse cells lines.

-anti-GPR116, Abcam cat. number ab111169, lot.GR48909-3,Validation: Tang X et al. GPR116, an adhesion G-protein-coupled receptor, promotes breast cancer metastasis via the Gaq-p63RhoGEF-Rho GTPase pathway. Cancer Res 73:6206-18 (2013). and also in this paper we have validation data on mouse cells lines.

-anti-beta tubulin, Sigma cat,number T5201,Involvement of argonaute proteins in gene silencing and activation by RNAs complementary to a non-coding transcript at the progesterone receptor promoter. Chu Y Nucleic Acids Research 38(21), 7736-7748, (2010), Beta-tubulin binds Src homology 2 domains through a region different from the tyrosine-phosphorylated protein-recognizing site. Itoh T The Journal of Biological Chemistry 271(44), 27931-27935, (1996)

-Recombinant Anti-VCP antibody [EPR3307(2)] (ab109240), Pinner AL et al. Protein expression of prenyltransferase subunits in postmortem schizophrenia dorsolateral prefrontal cortex. Transl Psychiatry 10:3 (2020).

Zhao Z et al. CB-5083, an inhibitor of P97, suppresses osteosarcoma growth and stem cell properties by altering protein homeostasis. Am J Transl Res 12:2956-2967 (2020).

Kaneko T et al. Identification and characterization of a large family of superbinding bacterial SH2 domains. Nat Commun 9:4549 (2018).

-anti-beta actin Sigma Adlrich Cat.# A5441, Validation: check references on the suppliers webpage: https://www.sigmaaldrich.com/catalog/product/sigma/a5441?lang=de&region=DE&cm_sp=Insite-_-caSrpResults_srpRecs_srpModel_a5441-_-srpRecs3-1

-anti-pAS160(Thr642) Cell Signaling Technology Cat.# 4288, Validation : Larance, M., Ramm, G., Stockli, J., van Dam, E. M., Winata, S., Wasinger,V., Simpson, F., Graham, M., Junutula, J. R., Guilhaus, M., and James, D. E.(2005)J. Biol. Chem.280,37803–37813

1. Watson, R.T. and Pessin, J.E. (2006) Trends Biochem. Sci. 31, 215-22.
2. Kane, S. et al. (2002) J. Biol. Chem. 277, 22115-8.
3. Sano, H. et al. (2003) J. Biol. Chem. 278, 14599-602.
4. Karlsson HK et al. (2005) Diabetes 54, 1692–7
5. Ramm, G. et al. (2006) J. Biol. Chem. 281, 29174-80.
6. Kramer, H.F. et al. (2006) J. Biol. Chem. 281, 31478-85.

-anti-AS160 Cell Signaling Technology Cat.# 2670S, validation: • Kane, S. et al. (2002) J. Biol. Chem. 277, 22115-8.
- Sano, H. et al. (2003) J. Biol. Chem. 278, 14599-602.
- Karlsson HK et al. (2005) Diabetes 54, 1692–7
- Ramm, G. et al. (2006) J. Biol. Chem. 281, 29174-80.
- Kramer, H.F. et al. (2006) J. Biol. Chem. 281, 31478-85.

-anti-AKT Cell Signaling Technology Cat.# 9272, 1. Validation : Franke, T.F. et al. (1997) Cell 88, 435-7.
2. Burgering, B.M. and Coffer, P.J. (1995) Nature 376, 599-602.
3. Franke, T.F. et al. (1995) Cell 81, 727-36.
4. Alessi, D.R. et al. (1996) EMBO J 15, 6541-51.
5. Sarbassov, D.D. et al. (2005) Science 307, 1098-101.
6. Jacinto, E. et al. (2006) Cell 127, 125-37.
7. Cardone, M.H. et al. (1998) Science 282, 1318-21.
8. Brunet, A. et al. (1999) Cell 96, 857-68.
9. Zimmermann, S. and Moelling, K. (1999) Science 286, 1741-4.
10. Cantley, L.C. and Neel, B.G. (1999) Proc Natl Acad Sci USA 96, 4240-5.
11. Vlahos, C.J. et al. (1994) J Biol Chem 269, 5241-8.
12. Hajduch, E. et al. (2001) FEBS Lett 492, 199-203.
13. Cross, D.A. et al. (1995) Nature 378, 785-9.
14. Diehl, J.A. et al. (1998) Genes Dev 12, 3499-511.
15. Gesbert, F. et al. (2000) J Biol Chem 275, 39223-30.
16. Zhou, B.P. et al. (2001) Nat Cell Biol 3, 245-52.
17. Navé, B.T. et al. (1999) Biochem J 344 Pt 2, 427-31.
18. Inoki, K. et al. (2002) Nat Cell Biol 4, 648-57.
19. Manning, B.D. et al. (2002) Mol Cell 10, 151-62.

-anti-phopsho-PKA substrate Cell Signaling Technology Cat.# 9624, Validation
1. Montminy, M. (1997) Annu Rev Biochem 66, 807-22.
2. Pearson, R.B. and Kemp, B.E. (1991) Methods Enzymol 200, 62-81.

-anti-pCREB(Ser133) Cell Signaling Technology Cat.# 9198, Validation:
1. Lonze, B.E. et al. (2002) Neuron 34, 371-85.
2. Lee, M.M. et al. (1999) J Neurosci Res 55, 702-12.
3. Redmond, L. et al. (2002) Neuron 34, 999-1010.
4. Dash, P.K. et al. (1990) Nature 345, 718-21.
5. Yin, J.C. et al. (1994) Cell 79, 49-58.
6. Guzowski, J.F. and McGaugh, J.L. (1997) Proc Natl Acad Sci USA 94, 2693-8.
7. Xing, J. et al. (1998) Mol Cell Biol 18, 1946-55.
8. Ribar, T.J. et al. (2000) J Neurosci 20, RC107.
9. Tan, Y. et al. (1996) EMBO J 15, 4629-42.
10. Devi, L. and Ohno, M. (2015) Transl Psychiatry 5, e562.

-anti-pERK1/2 (T202/Y204) Cell Signaling Technology , cat.no 9101 lot.28, Validation
1. Roux, P.P. and Blenis, J. (2004) Microbiol Mol Biol Rev 68, 320-44.
2. Baccarini, M. (2005) FEBS Lett 579, 3271-7.
3. Meloche, S. and Pouysségur, J. (2007) Oncogene 26, 3227-39.
4. Roberts, P.J. and Der, C.J. (2007) Oncogene 26, 3291-310.
5. Rubinfeld, H. and Seger, R. (2005) Mol Biotechnol 31, 151-74.
6. Murphy, L.O. and Blenis, J. (2006) Trends Biochem Sci 31, 268-75.
7. Dalby, K.N. et al. (1998) J Biol Chem 273, 1496-505.
8. Marais, R. et al. (1993) Cell 73, 381-93.
9. Kortenjann, M. et al. (1994) Mol Cell Biol 14, 4815-24.
10. Owens, D.M. and Keyse, S.M. (2007) Oncogene 26, 3203-13.
-anti-pAMPKa(Thr172) Cell Signaling Technology Cat.#2535, Validation:
1. Hardie, D.G. (2004) J Cell Sci 117, 5479-87.
2. Carling, D. (2004) Trends Biochem Sci 29, 18-24.
3. Hawley, S.A. et al. (1996) J Biol Chem 271, 27879-87.
4. Lizcano, J.M. et al. (2004) EMBO J 23, 833-43.
5. Shaw, R.J. et al. (2004) Proc Natl Acad Sci USA 101, 3329-35.
6. Woods, A. et al. (2003) J Biol Chem 278, 28434-42.
7. Warden, S.M. et al. (2001) Biochem J 354, 275-83.
8. Kim, E.K. et al. (2004) J Biol Chem 279, 19970-6.
9. Hadad, S.M. et al. (2009) BMC Cancer 9, 307.
-Phospho-Stat3 (Tyr705) (D3A7) XP® Rabbit mAb #9145 , Validation :
1. Heim, M.H. (2001) J Recept Signal Transduct Res 19, 75-120.
2. Takeda, K. et al. (1997) Proc Natl Acad Sci U S A 94, 3801-4.
3. Catlett-Falcone, R. et al. (1999) Immunity 10, 105-15.
4. Garcia, R. and Jove, R. (1998) J Biomed Sci 5, 79-85.
5. Bromberg, J.F. et al. (1999) Cell 98, 295-303.
6. Darnell, J.E. et al. (1994) Science 264, 1415-21.
7. Ihle, J.N. (1995) Nature 377, 591-4.
8. Wen, Z. et al. (1995) Cell 82, 241-50.
9. Yokogami, K. et al. (2000) Curr Biol 10, 47-50.
10. Biethahn, S. et al. (1999) Exp Hematol 27, 885-94.
-anti-HO-1 Cell Signaling Technology Cat.# 43966, Validation :
Abraham, N.G. and Kappas, A. (2008) Pharmacol Rev 60, 79-127.
Otterbein, L.E. et al. (2003) Trends Immunol 24, 449-55.
Cruse, I. and Maines, M.D. (1988) J Biol Chem 263, 3348-53.
Maines, M.D. (1988) FASEB J 2, 2557-68.
Schipper, H.M. et al. (2009) Curr Alzheimer Res 6, 424-30.
-pStat3 (phospho Y705) cell signalling cat.no. 9145, Validation :

Heim, M.H. (2001) J Recept Signal Transduct Res 19, 75-120.
Takeda, K. et al. (1997) Proc Natl Acad Sci U S A 94, 3801-4.
Catlett-Falcone, R. et al. (1999) Immunity 10, 105-15.
Garcia, R. and Jove, R. (1998) J Biomed Sci 5, 79-85.
Bromberg, J.F. et al. (1999) Cell 98, 295-303.
Darnell, J.E. et al. (1994) Science 264, 1415-21.
Ihle, J.N. (1995) Nature 377, 591-4.
Wen, Z. et al. (1995) Cell 82, 241-50.
Yokogami, K. et al. (2000) Curr Biol 10, 47-50.
Biethahn, S. et al. (1999) Exp Hematol 27, 885-94.
-anti-STAT3 Cell Signaling Technology Cat.#12640, Validation :
1. Heim, M.H. (2001) J Recept Signal Transduct Res 19, 75-120.
2. Takeda, K. et al. (1997) Proc Natl Acad Sci U S A 94, 3801-4.
3. Catlett-Falcone, R. et al. (1999) Immunity 10, 105-15.
4. Garcia, R. and Jove, R. (1998) J Biomed Sci 5, 79-85.
5. Bromberg, J.F. et al. (1999) Cell 98, 295-303.
6. Darnell, J.E. et al. (1994) Science 264, 1415-21.
7. Ihle, J.N. (1995) Nature 377, 591-4.
8. Wen, Z. et al. (1995) Cell 82, 241-50.
9. Yokogami, K. et al. (2000) Curr Biol 10, 47-50.
10. Biethahn, S. et al. (1999) Exp Hematol 27, 885-94.
-anti-phopsho-PKC substrate Cell Signaling Technology Cat.#6967,Validation :
1. Montminy, M. (1997) Annu Rev Biochem 66, 807-22.
2.Pearson, R.B. and Kemp, B.E. (1991) Methods Enzymol 200, 62-81.
-anti-ERK1/2, Cell signaling ,cat, no 4695, lot. no 21, Validation: Roux, P.P. and Blenis, J. (2004) Microbiol Mol Biol Rev 68, 320-44.

1. Baccarini, M. (2005) FEBS Lett 579, 3271-7.
2. Meloche, S. and Pouysségur, J. (2007) Oncogene 26, 3227-39.
3. Roberts, P.J. and Der, C.J. (2007) Oncogene 26, 3291-310.
4. Rubinfeld, H. and Seger, R. (2005) Mol Biotechnol 31, 151-74.
5. Murphy, L.O. and Blenis, J. (2006) Trends Biochem Sci 31, 268-75.
6. Dalby, K.N. et al. (1998) J Biol Chem 273, 1496-505.
7. Marais, R. et al. (1993) Cell 73, 381-93.
8. Kortenjann, M. et al. (1994) Mol Cell Biol 14, 4815-24.
9. Owens, D.M. and Keyse, S.M. (2007) Oncogene 26, 3203-13.
10. Suzuki, M. et al. (2015) J Immunol 195, 1273-81.

-anti-F(ab')2-Goat anti-Human IgG Fc Secondary Antibody, PE, Invitrogen, cat. no H10104, Validaton : Najjar AM,Manuri PR,Olivares S,Flores L,Mi T,Huls H,Shpall EJ,Champlin RE,Turkman N,Paolillo V,Roszik J,Rabinovich B,Lee DA,Alauddin M,Gelovani J,Cooper LJ Mol. Imaging Biology 2016

-polyclonal rabbit anti-CD68 antibody, #125212, Abcam, Validation for IHC.

Zhou YQ et al. Genetically engineered distal airway stem cell transplantation protects mice from pulmonary infection. EMBO Mol Med 12:e10233 (2020).

Zheng L et al. Inhibition of TIM-4 protects against cerebral ischaemia-reperfusion injury. J Cell Mol Med 24:1276-1285 (2020).

- Rabbit IgG, polyclonal - Isotype Control (ChIP Grade) (ab171870) abcam Cat.# 171870, Validation: Wang R & Huang K CCL11 increases the proportion of CD4+CD25+Foxp3+ Treg cells and the production of IL-2 and TGF-ß by CD4+ T cells via the STAT5 signaling pathway. Mol Med Rep 21:2522-2532 (2020). We also have validation that it is working as a control for blocking antibody in Fig 4 k (this paper).

-anti-rabbit, HRP conjugated , DAKO, P0399, Validation :

Neubert, P., Homann, A., et al..PlosBiology 2020,

Bettoni, S., Shaughnessy, J., et al JCI insight; 2019

-anti-mouse, HRP conjugated, DAKO, P0447, Validation :

Sampath Kumar Vemula, Ayse Malci1,Lennart Junge, Anne-Christin Lehmann,Ramya Rama, Johannes Hradsky, Ricardo A. Matute, André Weber,Matthias Prigge, Michael Naumann, Michael R. Kreutz,Constanze I. Seidenbecher, Eckart D. Gundelfinger and Rodrigo Herrera-Molina; Front. InCellAndDev.Biol.; Dec2020

Milena Denkiewicz-Kruk, Malgorzata Jedrychowska, Shizuko Endo2, Hiroyuki Araki,Piotr Jonczyk, Michal Dmowski,*and Iwona J. Fijalkowska; Int.J.f Mol.Sc.; Dec.2020

-secondary antibody: goat anti-rabbit IgG antibody (H+L), biotinylated, BA-1000, Vector, Germany, Validation:Yip SH,Romanò N,Gustafson P,Hodson DJ,Williams EJ,Kokay IC,Martin AO,Mollard P,Grattan DR,Bunn SJ. Elevated Prolactin during Pregnancy Drives a Phenotypic Switch in Mouse Hypothalamic Dopaminergic Neurons. Cell Rep.2019

- We have validated the Elisa by testing detection of purifiedFNDC4 recombinant protein and sepcificity in AAVshFNDC4 induction deletion of FNDC4 in mice. In addition we tested for cross reactivity to the close homolog Irisin and we found the Elisa to to be specifically detecting FNDC4.

-Phospho-Akt (Ser473) Antibody, Cell Signaling Technology Cat.# 9271S,

Franke, T.F. et al. (1997) Cell 88, 435-7.
Burgering, B.M. and Coffer, P.J. (1995) Nature 376, 599-602.
Franke, T.F. et al. (1995) Cell 81, 727-36.
Alessi, D.R. et al. (1996) EMBO J 15, 6541-51.
Sarbassov, D.D. et al. (2005) Science 307, 1098-101.
Jacinto, E. et al. (2006) Cell 127, 125-37.
Cardone, M.H. et al. (1998) Science 282, 1318-21.
Brunet, A. et al. (1999) Cell 96, 857-68.
Zimmermann, S. and Moelling, K. (1999) Science 286, 1741-4.
Cantley, L.C. and Neel, B.G. (1999) Proc Natl Acad Sci USA 96, 4240-5.
Vlahos, C.J. et al. (1994) J Biol Chem 269, 5241-8.
Hajduch, E. et al. (2001) FEBS Lett 492, 199-203.
Cross, D.A. et al. Nature 378, 785-9.
Diehl, J.A. et al. (1998) Genes Dev 12, 3499-511.
Gesbert, F. et al. (2000) J Biol Chem 275, 39223-30.
Zhou, B.P. et al. (2001) Nat Cell Biol 3, 245-52.
Navé, B.T. et al. (1999) Biochem J 344 Pt 2, 427-31.
Inoki, K. et al. (2002) Nat Cell Biol 4, 648-57.
Manning, B.D. et al. (2002) Mol Cell 10, 151-62.
Devi, L. and Ohno, M. (2015) Transl Psychiatry 5, e562.

Eukaryotic cell lines

Policy information about cell lines

Cell line source(s)	NIH3T3 cells were purchased from ATCC, imm. SVF cells lines have been previously used by Wu J et al; Cell 2012 and available in ATCC. The specific subpopulations sorted (HBC, LBC) here will be submitted to ATCC and are available from the authors upon request. HEK293A, HEK293T, 3T3L1, CHOS cells are available from ATCC.
Authentication	None of the cell line were authenticated.

Mycoplasma contamination	Mostly cell lines have been tested for mycoplasma contamination and tests in cell culture cells lines take place regularly in our lab and have returned negative results for mycoplasma contamination.
Commonly misidentified lines (See ICLAC register)	HepG2; We used it as a hepatocyte cell lines. No contamination with other type of cell lines has been used, however it has been described that not all HepG2 cell lines may be originating form the same donor.

Animals and other organisms

Policy information about studies involving animals; ARRIVE guidelines recommended for reporting animal research

Laboratory animals	- For studies with recombinant protein injections we used Wild type -genotype mice (Mus musculus) from Charles Rivers supplier, C57Bl6N genetic background. We used male mice, 12-25 weeks old.<br>-For isolation of primary SVF cells we used Wild type mice, 6-7weeks old, males, purchased by Charles Rivers laboratories.<br>- GPR116Ad-/- (adiponectinCrexGPR116 flox/flox mice), were derived from breedings of the Adiponectin Cre (C57Bl6Jgenetic background) to GPR116flox/flox (C57BL6N genetic background). They on a C57bl6N/J mixed background. In the manuscript we used male mice, 20-22 weeks old.<br>- For isolation of primary SVF cells from GPR116 WT, HET, KO mice. Mice were on a C57Bl6N genetic background, males, 6-7weeks old.<br>- For isolation of primary islets, WT mice, male, 8-13 weeks old, C57Bl6N, from Charles Rivers were used.<br>All mice were kept in mouse animals facility within the institute, under controlled temperature, light and air humidity conditions. The rooms were mice were kept in had ambient temperature of 20-22oC, 46-65% relative humidity, appropriate for mice, on a 12-h light/dark cycle.
Wild animals	Our study did not include wild animals
Field-collected samples	Our study did not include field-collected samples

Human research participants

Policy information about studies involving human research participants

Population characteristics	- For the Cross sectional study (Leipzig) Paired samples of subcutaneous (SC), omental visceral adipose tissue (AT) and liver were obtained from 66 individuals (29 women, 37 men). Age range: 36-97. BMI ranged from 22.7 to 45.6 kg/m², with or without Type 2 diabetes.<br>- For the Weight loss intervention studies (Leipzig) Paired SC, omental visceral AT and serum samples were further collected from 50 morbidly obese individuals, 20males and 30females, with age range:28-65 in the context of a 2-step bariatric surgery approach. In parallel, other 50 individuals, 13 males and 34 females, with age range: 44-58 underwent a combined exercise and diet intervention program over 12 months.<br>- The NUGAT feeding study included 92 healthy, non-diabetic, non-obese subjects, 34 males and 58 females (46 pairs of twins – 34 monozygotic and 12 dizygotic). The age ranged from 18 to 70 with a median of 25.
Recruitment	- For the cross sectional and Weight loss intervention studies participants were patients in the Leipzig clinic. Carrying doctors collected information on anthropometric characteristics, medical history and the patients files were anonymized. A blinded researcher assigned the patients in groups based on the BMI (body weight/height^2) and categorized in group of non diabetic and T2D base on the following criteria: the requirement of anti-diabetic medications, HbA1c <6%, fasting plasma glucose <6.0 mmol/l.<br>- In the NUGAT feeding study exclusion criteria were consumptive diseases, diabetes mellitus, high-grade anemia, renal failure, moderate to severe heart diseases, angina pectoris, or stroke in the last 6 months, food allergy, eating disorders, body weight change ≥3 kg within 3 months prior to the study, pregnancy or breastfeeding, drugs influencing metabolic homeostasis, lipid and liver metabolism, or inflammation (eg, systemic corticosteroids). Participants were initially screened to determine their eligibility for enrollment in the intervention study. This screening visit comprised physical examination, medical history, anthropometric measurements, and blood analysis. Additionally, a standardized 3-hour, 75-g oral glucose tolerance test (OGTT) was performed. The dietary intervention was carried out in a sequential design and under isocaloric conditions. Individual energy requirements were calculated based on participants resting energy expenditure (REE) determined by indirect calorimetry and physical activity level assessed by questionnaire. Participants were standardized for their nutritional behavior prior to the study via a 6-week carbohydrate-rich low-fat diet (LF, 55% carbohydrate, 30% fat, 15% protein) before they switched to a 6-week HF diet (40% carbohydrate, 45% fat, 15% protein) with emphasis on foods high in saturated fat 41. Participants were given intensive, regular and detailed dietary guidance by a nutritionist over the entire period of intervention to ensure compliance. During HF diet intervention fasting glucose values and glucose tolerance were assessed by intravenous GTT as well as fasting insulin values and HOMA-IR.<br>In all studies the data were gathered from many independent doctors and researchers, which were all blinded and working with anonymized patient folders.

Flow Cytometry

Plots

Confirm that:

- [x] The axis labels state the marker and fluorochrome used (e.g. CD4-FITC).
- [x] The axis scales are clearly visible. Include numbers along axes only for bottom left plot of group (a 'group' is an analysis of identical markers).
- [x] All plots are contour plots with outliers or pseudocolor plots.
- [x] A numerical value for number of cells or percentage (with statistics) is provided.

Methodology

Sample preparation	This is described under Methods : "FACS binding assay and sorting´´
Instrument	BD FACS Canto II and BD FACS Aria III from Biorad
Software	BD FACS Diva
Cell population abundance	Cell lines were used. 10000cells from the total cells was recorded in all experiments. Gating of PE positive cells versus background PE is described in supplementary.
Gating strategy	Supplementary information is provided to describe gating. Shortly staining with only secondary IgG PE was used to determine background signal . To assess the degree of binding of FcsFNDC4 on the examined cells we selected all cells and therefore the analysis was performed in the total populations of cells, without using gating to select subpopulations of cells. When we sorted for FcsFNDC4 negative and positive binding to cells (HBC and LBC) we did use gating to define and sort out these subpopulations.

- [x] Tick this box to confirm that a figure exemplifying the gating strategy is provided in the Supplementary Information.
